# Theoretical Prediction and Explanation of Reaction Site Selectivity in the Addition of a Phenoxy Group to Perfluoropyrimidine, Perfluoropyridazine, and Perfluoropyrazine

**DOI:** 10.3390/molecules26247637

**Published:** 2021-12-16

**Authors:** Timothy J. Fuhrer, Matthew Houck, Rachel M. Chapman, Scott T. Iacono

**Affiliations:** 1 Department of Chemistry, Radford University, Radford, VA 24142, USA; rchapman5@radford.edu; 2 Chemistry Research Center, Department of Chemistry, United States Air Force Academy, Colorado Springs, CO 80840, USA; houck308@gmail.com (M.H.); scott.iacono@afacademy.af.edu (S.T.I.)

**Keywords:** fluorine, diazine, aromatic, density functional theory

## Abstract

Perfluoroaromatics, such as perfluoropyridine and perfluorobenzene, are privileged synthetic scaffolds in organofluorine methodology, undergoing a series of regioselective substitution reactions with a variety of nucleophiles. This unique chemical behavior allows for the synthesis of many perfluoroaromatic derived molecules with unique and diverse architectures. Recently, it has been demonstrated that perfluoropyridine and perfluorobenzene can be utilized as precursors for a variety of materials, ranging from high performance polyaryl ethers to promising drug scaffolds. In this work, using density functional theory, we investigate the possibility of perfluoropyrimidine, perfluoropyridazine, and perfluoropyrazine participating in similar substitution reactions. We have found that the first nucleophilic addition of a phenoxide group substitution on perfluoropyrimidine and on perfluoropyridazine would happen at a site para to one of the nitrogen atoms. While previous literature points to mesomeric effects as the primary cause of this phenomenon, our work demonstrates that this effect is enhanced by the fact that the transition states for these reactions result in bond angles that allow the phenoxide to π-complex with the electron-deficient diazine ring. The second substitution on perfluoropyrimidine and on perfluoropyridazine is most likely to happen at the site para to the other nitrogen. The second substitution on perfluoropyrazine is most likely to happen at the site para to the first substitution. The activation energies for these reactions are in line with those reported for perfluoropyridine and suggest that these platforms may also be worth investigation in the lab as possible monomers for high performance polymers.

## 1. Introduction

Perfluoroaromatics are a privileged scaffold in organofluorine chemistry, often used in the production of various drugs, agrochemicals, and high-performance fluoropolymers [1,2,3]. Fluoropolymers, particularly those with fluoroaromatics in their architectures, are widely known for their desirable properties such as chemical resistance, thermal stability, and solution and melt processability [3,4,5,6]. Fluoroaromatics have also shown great promise for use in biomedical applications [7,8,9,10], including as substituents to allow medications to more easily cross the blood brain barrier [11,12] and penetrate cells [13].

**Figure 1:**
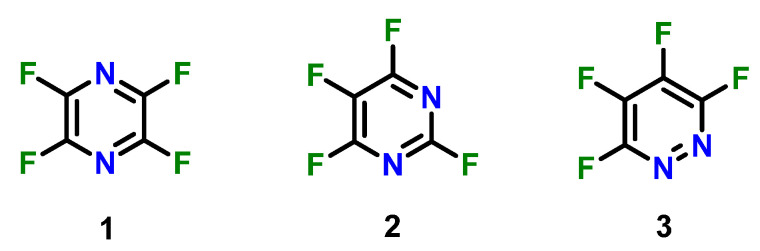
Perfluoropyrazine (**1**), perfluoropyrimidine (**2**), and perfluoropyridazine (**3**).

The chemistry of selected systems such as perfluoropyridine is now well established by experiment and supported by theory, demonstrating that these systems can undergo a series of regioselective substitution reactions with a variety of nucleophiles owing to its unique electronic structure [14]. Previous work has provided experimental and theoretical explanations of reaction site selectivity for this system and has also shed light on the significance of the perfluorination effect, caused by the inductive effect of fluorine that withdraws the electron density, leaving a region of diminished negative charge (a “hole”) that can then give rise to noncovalent interaction, which can have a significant impact on chemical reactivity. The perfluorination effect in hexafluorobenzene is experimentally well documented, with one of the more striking examples of the effect being the formation of a solid from the 1:1 mixture of hexafluorobenzene with mesitylene, both of which are liquids at room temperature [15]. Other work has also shed light on the perfluorination effect in perfluoropyridine and how it enables significant non-covalent interactions, which was recently shown to enhance the S_N_Ar substitution of perfluoropyridine [16,17]. The theoretical study of how these interactions affect chemical reaction outcomes and transition state geometries during the S_N_Ar substitution of perfluoroaromatics remains sparse. In the pursuit of other potential fluorinated scaffolds with unique chemical properties, diazaperfluoro-heteroaromatics such as perfluoropyrazine (**1**), perfluoropyrimidine (**2**), and perfluoropyridazine (**3**) are immediately attractive targets for chemical modeling (Figure 1) [18,19,20,21]. These molecules have been of increasing significance in the literature over the last several decades [22].

Given the significant fluorine content of these systems, they also appear to be excellent models for further testing the perfluorination effect and its outcome on chemical reaction processes. Examination of the reactions of molecules **1–3** with a phenoxide ion was established with regio-selectivity in the first step. The reaction of each of those products with another phenoxide was then determined with region-selectivity in the second step. Our goal was to examine reactants and possible products, intermediates, and transition states to determine which products were most likely to form. We assumed, as we did in our perfluoropyridine work [23], the reactions proceed without formation of a Meisenheimer intermediate. Some reports debate whether these reactions should be considered as two-step processes with a Meisenheimer intermediate, or as a concerted reaction with a short-lived transition state [24,25]. Recent work suggests that the answer to this question depends on the quality of the leaving group. Poor leaving groups, such as fluoride ion in the cases being discussed here, are more likely to cause a concerted reaction with a single transition state rather than a Meisenheimer intermediate [26]. This work demonstrates an in-depth study of simulated regio-selective additions of phenoxide additions to perfluoroaromatics **1–3** and their relative activation energies as potentially attractive scaffolds for the preparation of molecules of complex architectures. 

## 2. Results

### 2.1. First Phenoxide Substitution

**Figure 2:**
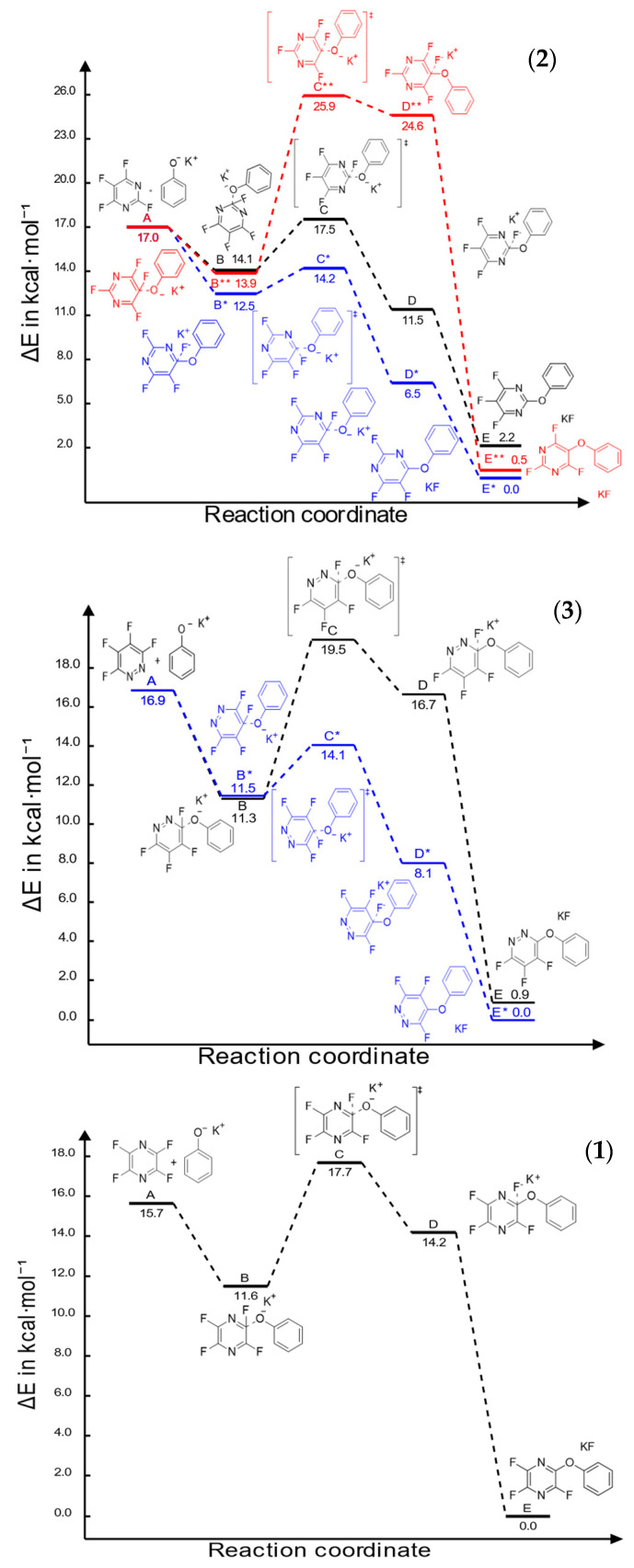
Reaction coordinate diagram for the first phenoxide addition to perfluoropyrimidine (**2**), perfluoropyridazine (**3**), and perfluoropyrazine (**1**).

For the cases of perfluoropyrimidine (**2**) and perfluoropyridazine (**3**), the carbon at site number four, para to one of the nitrogen atoms, was the most likely to undergo nucleophilic aromatic substitution with a phenoxide group. This outcome is based on electronic as well as kinetic factors, particularly the energy level of the transition state, rather than thermodynamic factors, in that the products have nearly the same energy in both cases (Figure 2). Perfluoropyrazine’s symmetry only allows for one unique reaction site whichwas modelled only for the sake of comparing reactivity via activation energy.

The activation energies for the phenoxide substitutions are compared in Table 1, the exception of which is associated with perfluoropyrazine, whose activation energy is 6.167 kcal/mol (Figure 1). This is significantly larger than that of the reactions at carbon four for perfluoropyrimidine (1.699 kcal/mol) and perfluoropyridazine (2.572 kcal/mol). For the sake of comparison, the previously reported computed activation energy associated with a substitution at carbon four on perfluoropyridine is 4.16 kcal/mol [23]. It is thus expected that both perfluoropyrimidine and perfluoropyridazine will react faster with phenoxide ion than perfluoropyridine, while perfluoropyrazine will react more slowly.

**Figure 3:**
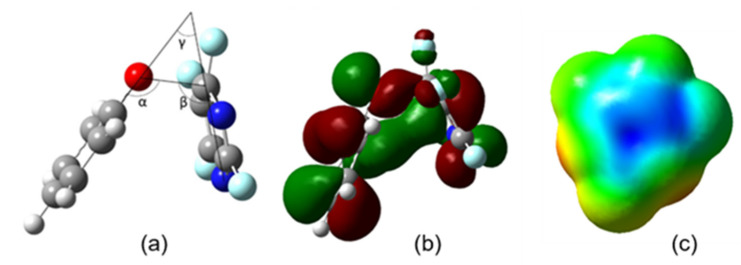
(**a**) Depiction of approach angles formed during reaction of phenoxide and perfluoropyrimidine. The bond angles are labeled corresponding to the data in Table 1. (**b**) HOMO diagram showing π orbital interactions in reaction of phenoxide and perfluoropyrimidine at carbon 4. (**c**) Electrostatic potential map of perfluoropyrimidine showing the electron deficient “π-hole” at the center of the perfluoropyrimidine ring.

The question is, why is there such a stark difference between the transition state energies, and thus the activation energies (Table 1) for these reactions when they happen at different carbon atoms? Previous experimental work by Sandford et.al. [27,28] and Banks et.al. [29] suggests that mesomeric effects are very important to this discussion. However, in the cases in this study, where the substitution species is a phenoxide ion, these effects appear to be enhanced by an optimization of the angles between the planes defined by the aromatic rings (Figure 3). The transition state in Figure 3a defines an angle γ (the angle between the least-squares aromatic ring planes of the two reactants), an angle α (C_Ph_–O- –C4_diazine_F), and an angle β (N1_diazine_–C4_diazine_F–O).

The more acute the angle γ, the closer the two planes are to parallel, and the more efficient the π-complexation between them (Figure 3a,b). A scan of the energy surface of the transition state for phenoxide ion approaching at carbon four on perfluoropyrimidine (the example shown in Figure 3c) showed that opening this angle by fourteen degrees (the difference between the angle γ for the substitution transition state at carbon 4 and carbon 5) increased the energy of the system by 1 kcal/mol. The activation energy difference between those two is about 10.3 kcal/mol, about 1 kcal of which appears to be π-complexation energy. A scan of the energy surface of the transition state for phenoxide ion to be substituted at carbon four on perfluoropyridazine showed that opening this angle by four degrees (the difference between the angle γ for the substitution transition state at carbon 4 and carbon 3) increased the energy of the system by 0.1 kcal/mol. The activation energy difference between those two is about 5.6 kcal/mol, about 0.1 kcal of which appears to be π-complexation energy.

**Figure 4:**
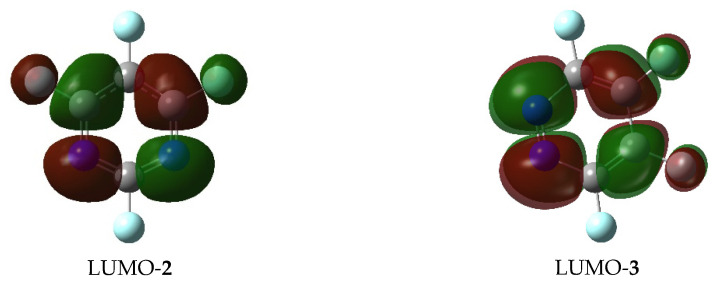
LUMO plots of **2** and **3** showing the lack of LUMO electron density around carbons 5 and 3.

Based on electrostatic density maps of the starting fluorodiazines, the observed face-to-face π-complexation is at least partially driven by the attraction of the electron-rich phenoxide ring to the electron deficient ring of the diazene, demonstrating the importance of the perfluorination effect in the formation of the transition state (Figure 3). It is worth noting that the geometries of the products do not retain the convex geometry of the two planes, indicating that the fluorine leaving group is conjugated with the ring and is necessary to maintain the stability of the π-complexation. Examination of the electrostatic density map of the substitution product shows that the newly formed aryl ether contributes significant electron density to the diazine ring, making face-to-face π-complexation less favorable. This coupled with the relatively extreme bond angles that would be required to maintain the convex geometry (or face-to-face orientation) in the transition structure help explain the geometric outcome of the product. Another contributor to the observed difference between the transition state energies could be the calculated low LUMO electron densities. In both perfluoropyrimidine (**2**) and perfluoropyridazine (**3**), a low degree of LUMO density is observed around carbons 5 and 3, respectively, the positions that are involved in the highest energy transition states for these systems. This is not surprising given that a low LUMO density should make the nucleophilic substitution more difficult, contributing to the sharp increase in the transition state energy (Figure 4).

### 2.2. Second Phenoxide Substitution

**Figure 5:**
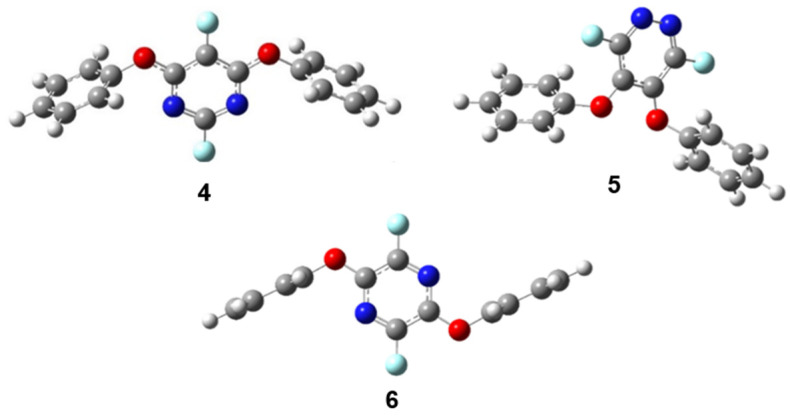
Products of second phenoxide substitutions: (**4**) second substitution product for perfluoropyrimidine, (**5**) second substitution product for perfluoropyridazine, (**6**) second substitution product for perfluoropyrazine.

A second phenoxide substitution was modeled for each perfluorodiazine and the most likely products are shown in Figure 5, starting with the lowest activation energy product for each. The activation energies are summarized in Table 2. Perfluoropyrimidine, when already substituted with a phenoxide group at carbon four, is most likely to undergo a second substitution at carbon six, the site meta to the original substitution, yielding symmetric product **4** with the dihedral angles between the pyrimidine ring and each of the phenoxide rings nearly perpendicular (Figure 5).

**Figure 6:**
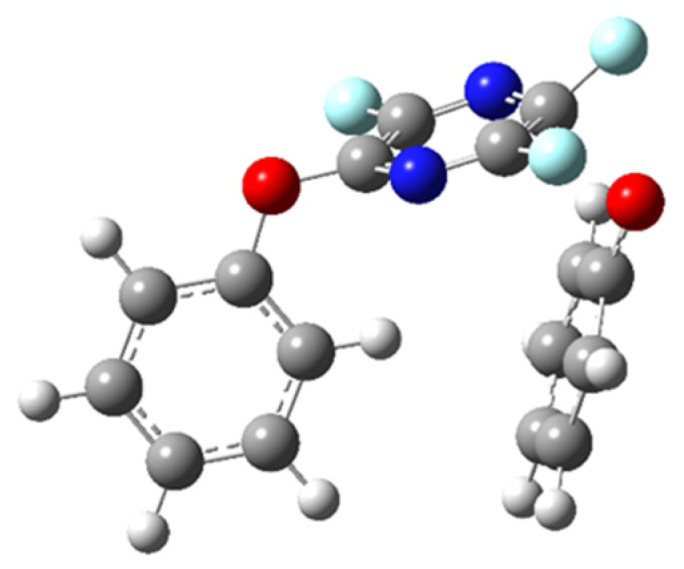
Transition state for second phenoxide substitution on perfluoropyrazine.

Perfluoropyridazine, when already substituted with a phenoxide group at carbon four, is most likely to undergo a second substitution at carbon five, ortho to the first substitution **5**. The two phenoxide planes are out of plane with one another, but not to the point of being orthogonal. Perfluoropyrazine, when already substituted with a phenoxide group at carbon four, is most likely to undergo a second substitution at carbon five, para to the first substitution with the two phenoxide planes parallel to each other and perpendicular to pyrazine ring **6** (Figure 5). We performed an angle analysis on the transition states for the second substitution with the trend relating angle γ to activation energy for the transition state that we noted for the first substitution holding for the second substitution as well, with the exception of perfluoropyrazine (Table 2). We have performed a thorough investigation of the potential energy surface for that particular transition state and have not been able to find a more stable structure than the one shown in Figure 6 for the addition of a phenoxide group to carbon five of 2-phenoxyperfluoropyrazine. This geometry appears to stem from the favorable T-shaped interaction between the electron-rich aromatic ring and the slightly electron-deficient hydrogen of the attacking phenoxide, making the perpendicular orientation of the nucleophile more favorable. While surprising, the T-shaped interaction between aromatic rings is well known and has been examined theoretically as well as observed experimentally [30,31,32].

## 3. Computational Methods

All models for this work were computed using the Gaussian 09 suite of programs, including use of Gaussview 5 to generate three-dimensional figures [33,34]. Each molecule to be modeled was constructed in the Arguslab [35] environment and had its geometry optimized first with molecular mechanics and then with the PM3 semi-empirical method. These structures were then used as starting structures for density functional theory (B3LYP/6-311G(D)). Geometries optimized with DFT were verified with frequency analysis at the same level of theory calculation as the optimization to assure no imaginary vibrational frequencies. Solvent effects of *N*,*N*-dimethylformamide were accounted for by the polarizable continuum model (PCM) [36,37,38], as implemented in Gaussian 09.

Reactants and products for each reaction (**A** and **E** in the energy level figures) were modeled by assuming separate molecules in solution. Reaction intermediates (**B** and **D** in the energy level figures) were first approximately modeled by the same method as the reactants and products, with starting geometries built from assuming the displacement of the fluoride ion in each case by the phenoxide ion. The intermediates on the reactants side were assumed to have the approximate geometries of having been brought near to each other in the solvent PCM. The intermediates on the products’ side were assumed to have the approximate geometries of the products with the fluoride ion still in close proximity to the carbon it was leaving and in the solvent PCM.

Transition states (**C** in the energy level figures) were modeled using the synchronous transit-guided quasi-Newton (STQN) method [39] to find the transition state between each pair of approximate intermediates. Each transition state model was then treated with frequency analysis to assure there was exactly one imaginary frequency and that imaginary frequency corresponded to movement between the two intermediates. After this, each transition state structure was analyzed by performing an intrinsic reaction coordinate (IRC) [40] analysis on the transition state model structures at the same level of theory at which the other models were performed. Once the most likely product was determined, models were constructed to determine at which site a second phenoxide substitution would take place. Note, the symmetry of **1** makes the first step in this process unnecessary.

To ensure that a global minimum rather than a local minimum is found for each species modeled, the potential energy surface for each optimized structure was scanned about each rotatable dihedral angle using the “modredundant” functionality in Gaussian 09. Lower energy geometry candidates were then optimized by the procedure above and the lowest overall energy geometry was chosen for each molecule. The “modredundat” feature in Gaussian 09 was also used to scan the potential energy surface as a means of determining the effect of opening the γ angle for the transition state structures. The reaction coordinate diagrams of second phenoxide substitutions and cartesian coordinates of all computed structures can be found in the Supplemental Materials.

## 4. Conclusions

We have shown, through the use of density functional theory, the likely products of a first and second phenoxide substitution on perfluoropyrimidine, perfluoropyridazine, and perfluoropyrazine. The transition states for each substitution seem to be at least partially stabilized by π-complexation between the substituting phenoxide ring and the electron deficient diazide ring, as seen in the observed approach angle trends. The activation energies for these reactions are in line with those reported for perfluoropyridine [23] and suggest that these platforms may also be worth investigation in the lab as possible monomers for high performance polymers. This section is not mandatory, but can be added to the manuscript if the discussion is unusually long or complex.

## Figures and Tables

**Table 1:** Angles formed in transition state of the reactant at specified carbon with phenoxide along with the calculated activation energy for formation.

Transition State	Angle α	Angle β	Angle γ	*E*_a_ (kcal/mol)
2-phenoxyperfluoropyrimidine	116.385	111.131	47.516	3.437
4-phenoxyperfluoropyrimidine	114.961	112.551	47.512	1.699
5-phenoxyperfluoropyrimidine	115.486	126.353	61.839	12.017
3-phenoxyperfluoropyridazine	116.108	118.859	54.967	8.137
4-phenoxyperfluoropyridazine	115.628	115.345	50.973	2.572

**Table 2:** Angles formed in transition involving the specified carbon atom of the substrate reacting with phenoxide ion and activation energy *E*_a_.

Transition State	Angle α	Angle β	Angle γ	*E*_a_ (kcal/mol)
2,4-phenoxyperfluoropyrimidine	117.838	113.505	51.343	5.945
4,5-phenoxyperfluoropyrimidine	115.658	126.230	61.888	15.945
4,6-phenoxyperfluoropyrimidine	115.536	115.234	50.770	3.937
3,4-phenoxyperfluoropyridazine	116.588	117.413	54.002	8.260
4,5-phenoxyperfluoropyridazine	115.849	114.699	50.549	2.600
4,6-phenoxyperfluoropyridazine	117.044	120.732	57.776	8.241
2,3-phenoxyperfluoropyrazine	116.616	116.291	52.907	6.270
2,5-phenoxyperfluoropyrazine	117.493	116.755	54.248	6.189
2,6-phenoxyperfluoropyrazine	115.942	119.439	55.381	8.310

## Data Availability

Not applicable.

## Supplementary Materials

The following are available online, Figures S1–S3: Reaction Coordinate Diagrams of Second Phenoxide Substitutions; Table S1: Cartesian Coordinates for Optimized Structures.

## Sample Availability

Samples of the compounds are not available from the authors.
