# Supplementary material for: Theoretical Prediction and Explanation of Reaction Site Selectivity in the Addition of a Phenoxy Group to Perfluoropyrimidine, Perfluoropyridazine, and Perfluoropyrazine
Source: Molecules. 2021 Dec 16;26(24):7637. doi: 10.3390/molecules26247637 (PMC8705331; doi:10.3390/molecules26247637)
Supplement: Supplementary file 1 [file molecules-26-07637-s001.zip › molecules-1507281-supplementary.pdf]

Theoretical Prediction and Explanation of Reaction Site Selectivity in the Addition of a Phenoxy Group to Perfluoropyrimidine, Perfluoropyridazine and Perfluoropyrazine

Timothy J. Fuhrer[1*†] Matthew Houck[2†], Rachel M. Chapman[1] and Scott T. Iacono[2]

1. Department of Chemistry, Radford University, Radford, Virginia
2. Department of Chemistry & Chemistry Research Center,
United States Air Force Academy, Colorado Springs, Colorado
†Co-first authors

*Correspondence e-mail: tfuhrer@radford.edu

SUPPLEMENTARY INFORMATION

Figure S1: Reaction energy diagram showing the possible paths taken during second phenoxide substitutions for perfluoropyrazine.

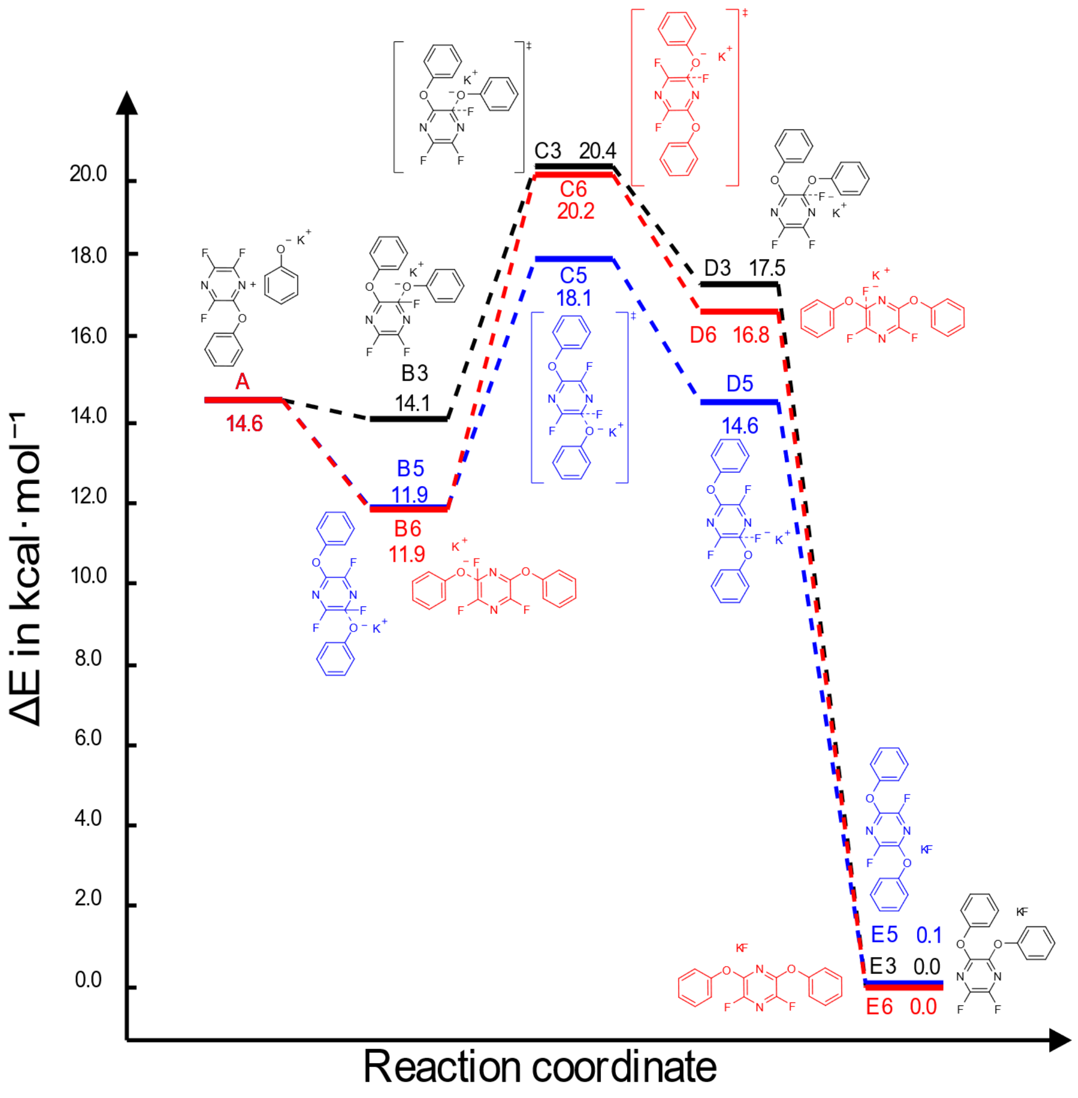

Figure S2: Reaction energy diagram showing the possible paths taken during second phenoxide substitutions for perfluoropyridazine.

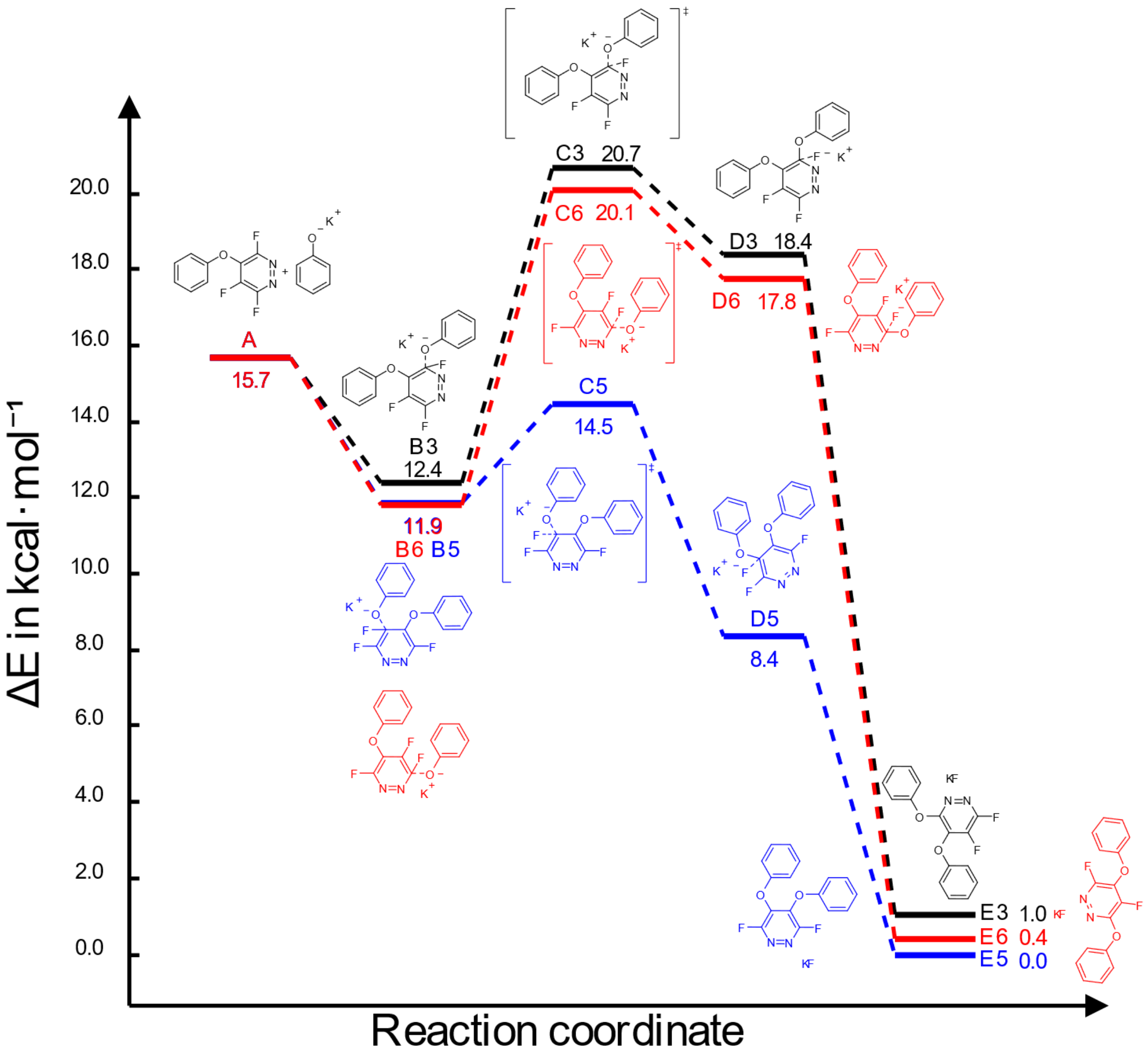

Figure S3: Reaction energy diagram showing the possible paths taken during second phenoxide substitutions for perfluoropyrimidine.

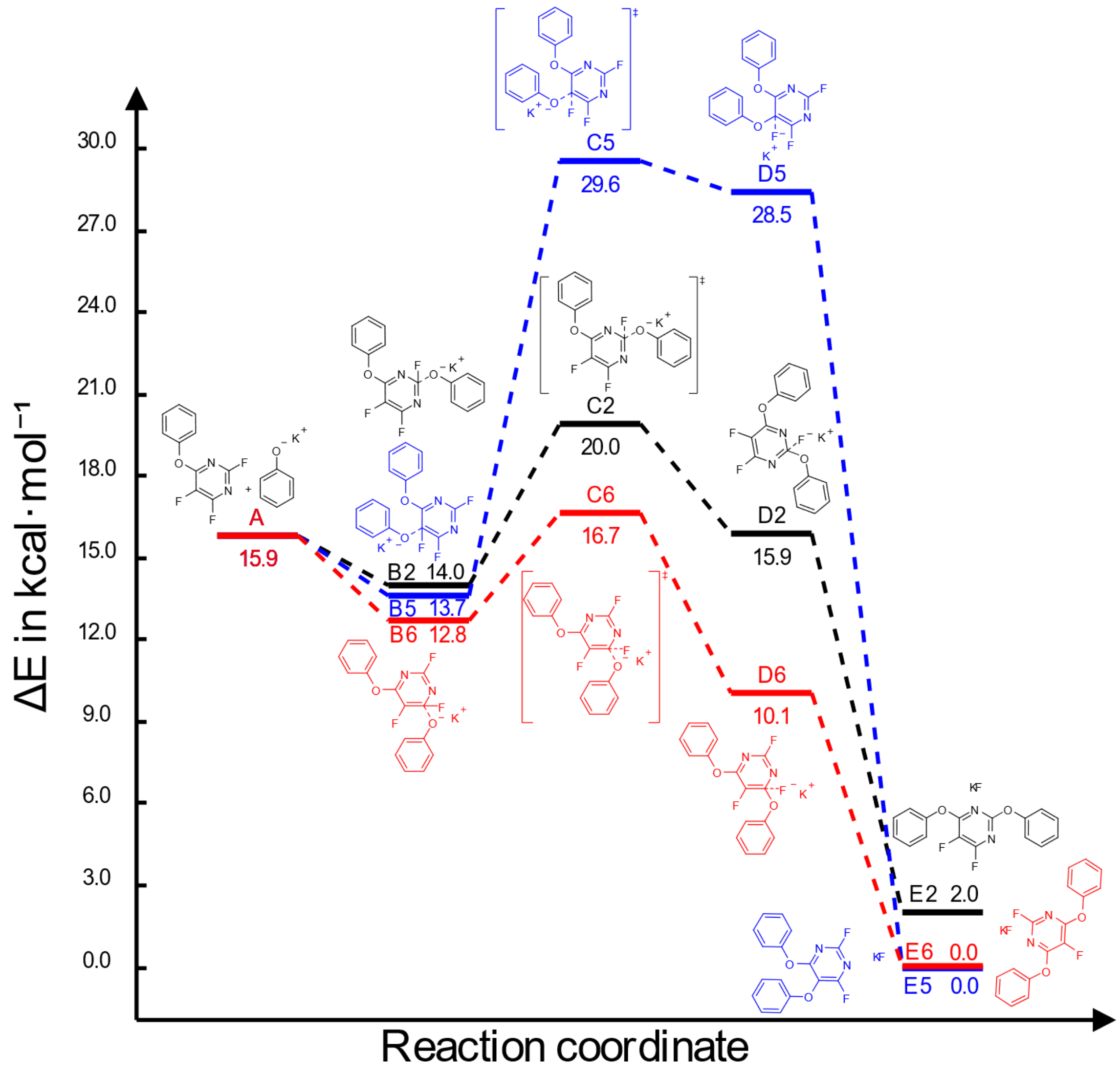

Table S1: CARTESIAN COORDINATES FOR OPTIMIZED STRUCTURES

33
3pyrazine2ndSub1stINT.xyz

C	-1.08900	2.17260	0.28630
N	-0.53160	1.65680	1.35770
C	0.32620	0.68560	1.17170
C	0.68200	0.23360	-0.10520
N	0.12080	0.77370	-1.17110
C	-0.76170	1.72670	-0.98540
O	1.54070	-0.80630	-0.28430
F	-1.98200	3.15190	0.45370
F	0.92390	0.17440	2.24480
C	-4.73510	-1.63960	-1.06640
C	-3.52660	-2.17400	-1.53150
C	-2.37260	-2.13180	-0.75870
C	-4.75130	-1.06210	0.20980
C	-3.60610	-1.01210	0.99490
C	-2.34460	-1.54580	0.55450
O	-1.28290	-1.50070	1.27060
H	-5.63370	-1.67680	-1.67470
H	-3.48750	-2.63060	-2.51930
H	-1.44300	-2.55240	-1.13760
H	-5.67860	-0.64150	0.59620
H	-3.63790	-0.56020	1.98470
C	5.08120	-1.64200	-0.29460
C	5.68790	-0.39640	-0.12110
C	4.89360	0.74060	0.00290
C	3.69530	-1.75070	-0.33970
C	2.91420	-0.60280	-0.21330
C	3.50140	0.64850	-0.04490
H	5.68820	-2.53550	-0.39450
H	6.76850	-0.31470	-0.08520
H	5.35240	1.71480	0.13470
H	3.20630	-2.70910	-0.47180
H	2.89680	1.54370	0.03770
F	-1.34040	2.26930	-2.06380

33
3pyrazine2ndSub2ndINT.xyz

C	-1.20260	2.11090	0.19590
N	-1.05690	1.30610	1.21700
C	-0.26210	0.18670	1.07310
C	0.47290	0.12770	-0.21240
N	0.28460	0.95830	-1.18950

C	-0.57550	1.95730	-1.01710
O	1.35540	-0.93310	-0.38600
F	-2.02620	3.17790	0.38300
F	0.68250	0.10590	2.15860
C	-4.42890	-1.88460	-0.83890
C	-3.18530	-2.33330	-1.28370
C	-2.03180	-2.04950	-0.55290
C	-4.51480	-1.15350	0.34670
C	-3.36560	-0.86520	1.08070
C	-2.11990	-1.31350	0.63190
O	-0.98910	-1.07130	1.37820
H	-5.32520	-2.10750	-1.40860
H	-3.11050	-2.90680	-2.20230
H	-1.05960	-2.39550	-0.88630
H	-5.47940	-0.80340	0.70090
H	-3.41540	-0.29300	1.99920
C	4.91880	-1.66140	-0.52600
C	5.49330	-0.44810	-0.14350
C	4.66410	0.62870	0.16530
C	3.53560	-1.79490	-0.60330
C	2.71270	-0.70920	-0.29210
C	3.27630	0.50830	0.09480
H	5.54940	-2.51070	-0.76900
H	6.57150	-0.34470	-0.08680
H	5.09560	1.57780	0.46760
H	3.07720	-2.73190	-0.90060
H	2.64190	1.34960	0.34120
F	-0.77580	2.81810	-2.06310

32
3pyrazine2ndSubPRODUCT.xyz

C	0.69020	2.43390	0.00150
N	1.37450	1.30850	0.00140
C	0.71000	0.17440	-0.00080
C	-0.71010	0.17480	-0.00290
N	-1.37390	1.30920	-0.00270
C	-0.68900	2.43430	-0.00050
O	-1.33470	-1.02210	-0.00520
F	1.37980	3.58310	0.00380
C	5.49470	-1.31010	0.00060
C	4.79850	-1.25600	1.20800
C	3.40980	-1.13080	1.21340
C	4.80050	-1.24310	-1.20730
C	3.41180	-1.11790	-1.21370
C	2.73790	-1.06260	-0.00030
O	1.33410	-1.02280	-0.00080

H	6.57470	-1.40920	0.00100
H	5.33390	-1.31280	2.14960
H	2.85140	-1.09330	2.14180
H	5.33750	-1.28980	-2.14850
H	2.85500	-1.07050	-2.14250
C	-4.79790	-1.24470	1.20990
C	-5.49520	-1.30980	0.00370
C	-4.80220	-1.25340	-1.20550
C	-3.40930	-1.11930	1.21290
C	-2.73850	-1.06180	-0.00210
C	-3.41360	-1.12790	-1.21420
H	-5.33240	-1.29320	2.15240
H	-6.57520	-1.40910	0.00600
H	-5.34010	-1.30860	-2.14570
H	-2.85010	-1.07350	2.14040
H	-2.85760	-1.08850	-2.14400
F	-1.37790	3.58390	-0.00040

33
3TSpyrazine2ndSub.xyz

C	-1.21120	2.09710	0.22610
N	-0.85880	1.42420	1.29810
C	-0.09020	0.33860	1.12430
C	0.52870	0.15760	-0.16830
N	0.17270	0.86150	-1.20580
C	-0.71960	1.82610	-1.03020
O	1.42910	-0.87360	-0.33380
F	-2.05140	3.13910	0.40870
F	0.70790	0.03980	2.19660
C	-4.53370	-1.58710	-0.91960
C	-3.31700	-2.12070	-1.35210
C	-2.16460	-1.97200	-0.58530
C	-4.58110	-0.90580	0.29930
C	-3.43240	-0.75140	1.07080
C	-2.19240	-1.28450	0.64990
O	-1.09810	-1.15990	1.39500
H	-5.43110	-1.70780	-1.51790
H	-3.26720	-2.65800	-2.29550
H	-1.22040	-2.39350	-0.91650
H	-5.52200	-0.49030	0.65010
H	-3.46860	-0.22730	2.02060
C	5.00350	-1.54300	-0.43950
C	5.55470	-0.30200	-0.11470
C	4.70770	0.77420	0.14100
C	3.62350	-1.70560	-0.50920

C	2.78510	-0.61950	-0.25080
C	3.32190	0.62560	0.07580
H	5.64990	-2.39090	-0.64130
H	6.63070	-0.17710	-0.06210
H	5.12220	1.74410	0.39600
H	3.18030	-2.66310	-0.75970
H	2.67290	1.46930	0.27540
F	-1.08880	2.55420	-2.11530

33
5secondSub2ndIntPyrazine.xyz

N	0.34470	0.49370	-1.06100
C	-0.65920	1.27480	-0.95070
C	-1.41570	1.54390	0.29250
N	-0.85150	0.96400	1.40820
C	0.18740	0.17940	1.25160
C	0.81100	-0.10770	0.05990
O	1.80270	-1.07320	-0.06310
F	-1.13440	1.88250	-2.08090
F	-1.54270	2.96380	0.47350
C	-3.90060	-2.76070	-0.40110
C	-3.82260	-2.22680	0.88580
C	-3.45870	-0.89440	1.07470
C	-3.62050	-1.95150	-1.50210
C	-3.25750	-0.61760	-1.32020
C	-3.17480	-0.08750	-0.02980
O	-2.85570	1.24230	0.14630
H	-4.18340	-3.79830	-0.54470
H	-4.04300	-2.84960	1.74700
H	-3.38870	-0.46680	2.06760
H	-3.68380	-2.35780	-2.50660
H	-3.03900	0.02220	-2.16780
C	5.41050	-1.43200	-0.33100
C	5.85880	-0.11540	-0.20360
C	4.92800	0.90650	-0.02780
C	4.05090	-1.72080	-0.28340
C	3.12480	-0.68880	-0.10430
C	3.56070	0.63040	0.02190
H	6.12240	-2.23950	-0.46880
H	6.91930	0.10910	-0.24190
H	5.26250	1.93440	0.07140
H	3.68660	-2.73800	-0.38030
H	2.84230	1.43000	0.15480
F	0.67280	-0.38590	2.39210

33
5secondSubFirstIntPyrazine.xyz

N	0.30460	0.79660	-0.98030
C	-0.53180	1.77170	-0.77860
C	-0.95980	2.11920	0.50990
N	-0.53160	1.47710	1.56100
C	0.30510	0.47770	1.35080
C	0.74300	0.12300	0.08570
O	1.57290	-0.94380	-0.10280
F	-0.95890	2.47050	-1.83060
F	-1.74680	3.17340	0.69030
C	-3.55290	-3.22050	-0.26390
C	-3.66300	-2.46770	0.91210
C	-3.54090	-1.08350	0.89750
C	-3.32120	-2.53790	-1.46460
C	-3.19440	-1.15440	-1.49520
C	-3.29830	-0.34580	-0.31210
O	-3.18110	0.93250	-0.33480
H	-3.65090	-4.30160	-0.24610
H	-3.84620	-2.97460	1.85820
H	-3.63440	-0.51360	1.81990
H	-3.23490	-3.09980	-2.39330
H	-3.01680	-0.63970	-2.43760
C	5.10720	-1.75980	-0.42340
C	5.71990	-0.51260	-0.28720
C	4.93230	0.61860	-0.09000
C	3.72220	-1.87600	-0.36190
C	2.94790	-0.73430	-0.16140
C	3.54140	0.51850	-0.02670
H	5.70890	-2.64910	-0.57820
H	6.79970	-0.42550	-0.33530
H	5.39520	1.59400	0.01580
H	3.22990	-2.83660	-0.46350
H	2.94170	1.40790	0.12150
F	0.74110	-0.18770	2.42660

32
5secondsubPRODpyrazine.xyz

N	-1.17310	-0.71450	-0.00020
C	-0.01010	-1.31180	-0.00020
C	1.19900	-0.60870	-0.00010
N	1.17300	0.71460	0.00010
C	0.01010	1.31190	0.00020
C	-1.19900	0.60880	0.00010

O	-2.35620	1.30880	0.00020
F	0.00140	-2.65130	-0.00050
C	6.05670	0.61430	0.00010
C	5.43190	0.30370	1.20770
C	4.18180	-0.31380	1.21340
C	5.43200	0.30390	-1.20770
C	4.18190	-0.31370	-1.21360
C	3.57510	-0.61220	-0.00010
O	2.35620	-1.30880	-0.00020
H	7.03000	1.09280	0.00020
H	5.91630	0.53960	2.14910
H	3.68400	-0.56840	2.14220
H	5.91650	0.53980	-2.14890
H	3.68420	-0.56820	-2.14240
C	-5.43170	-0.30450	1.20770
C	-6.05670	-0.61440	-0.00000
C	-5.43220	-0.30310	-1.20770
C	-4.18160	0.31310	1.21360
C	-3.57510	0.61220	0.00010
C	-4.18210	0.31440	-1.21340
H	-5.91590	-0.54100	2.14900
H	-7.03000	-1.09290	-0.00010
H	-5.91680	-0.53850	-2.14900
H	-3.68360	0.56710	2.14240
H	-3.68460	0.56950	-2.14220
F	-0.00140	2.65130	0.00040

33
5TSpyrazineSecondSub.xyz

N	0.25000	-1.75010	-0.98780
C	1.51940	-1.58940	-1.08110
C	2.36490	-1.21270	0.02770
N	1.81290	-1.22920	1.24160
C	0.50070	-1.36700	1.31270
C	-0.31230	-1.62770	0.23670
O	-1.65860	-1.89600	0.37830
F	2.10190	-1.73690	-2.29150
F	3.64800	-1.68110	-0.02810
C	0.36880	3.67740	0.37070
C	1.07850	3.05950	1.40270
C	1.92160	1.98200	1.14190
C	0.51410	3.19820	-0.93410
C	1.34520	2.11410	-1.20190
C	2.08070	1.48260	-0.17190
O	2.90480	0.47710	-0.44420

H	-0.27940	4.52300	0.57740
H	0.97820	3.42500	2.42130
H	2.48340	1.51300	1.94220
H	-0.02790	3.67170	-1.74830
H	1.46200	1.74180	-2.21560
C	-4.92180	-0.38810	-0.18130
C	-4.59400	0.91600	-0.55750
C	-3.25360	1.28910	-0.62610
C	-3.92140	-1.30660	0.12030
C	-2.58130	-0.91970	0.04910
C	-2.23860	0.37910	-0.32540
H	-5.96120	-0.69430	-0.12270
H	-5.37480	1.63080	-0.79390
H	-2.98360	2.29940	-0.91600
H	-4.15880	-2.32320	0.41410
H	-1.20100	0.68390	-0.38570
F	-0.03560	-1.29150	2.55120

22

TFPyridazine4TS.xyz

N	1.37644	-0.56744	1.85405
C	1.47447	-1.15580	0.70932
C	1.39842	-0.54256	-0.58000
C	1.41561	0.85075	-0.48282
C	1.28983	1.42477	0.77280
N	1.27179	0.78405	1.91507
F	1.61051	-2.49694	0.72401
F	2.03587	-1.13716	-1.61506
F	1.45393	1.60894	-1.59967
F	1.20918	2.76628	0.84580
C	-3.70859	0.46649	0.46054
C	-2.92716	1.22740	-0.41107
C	-1.77744	0.69245	-0.98809
C	-3.32004	-0.84544	0.74935
C	-2.16955	-1.38489	0.18399
C	-1.37015	-0.63730	-0.71636
H	-4.60567	0.88561	0.90502
H	-3.21696	2.24841	-0.64528
H	-1.18003	1.28369	-1.67527
H	-3.91771	-1.44918	1.42726
H	-1.86510	-2.40335	0.40879
O	-0.29626	-1.16585	-1.27326

6secondsubPRODpyrazine.xyz

N	-3.56200	-0.00130	0.00090
C	-2.88640	1.11950	0.01580
C	-1.48740	1.13820	0.01430
N	-0.81470	-0.00010	-0.00120
C	-1.48650	-1.13900	-0.01550
C	-2.88550	-1.12150	-0.01500
O	-0.86400	-2.33760	-0.03310
F	-3.57550	2.26890	0.03280
C	3.29190	2.65570	-0.04230
C	2.57760	2.57890	-1.23790
C	1.19020	2.44250	-1.22010
C	2.61720	2.59930	1.17700
C	1.22980	2.46250	1.20660
C	0.53770	2.38520	0.00490
O	-0.86590	2.33740	0.03050
H	4.37090	2.76350	-0.06100
H	3.09790	2.62690	-2.18830
H	0.61830	2.38810	-2.13930
H	3.16820	2.66310	2.10900
H	0.68760	2.42280	2.14430
C	2.61920	-2.60830	-1.17600
C	3.29380	-2.65380	0.04380
C	2.57930	-2.56680	1.23860
C	1.23170	-2.47210	-1.20690
C	0.53940	-2.38460	-0.00610
C	1.19180	-2.43130	1.21940
H	3.17030	-2.68000	-2.10740
H	4.37280	-2.76120	0.06350
H	3.09950	-2.60640	2.18940
H	0.68950	-2.44030	-2.14500
H	0.61990	-2.36920	2.13820
F	-3.57350	-2.27160	-0.03110

33

6SecondSubSecondINTpyrazine.xyz

N	-1.06280	-1.88720	1.47870
C	-1.50400	-1.78950	0.27940
C	-0.86310	-1.02540	-0.80600
N	0.19570	-0.26510	-0.38750
C	0.65350	-0.40690	0.85040
C	0.05430	-1.21680	1.78410
O	1.72150	0.37880	1.25050
F	-2.65300	-2.45810	-0.03520
F	-0.43220	-1.99660	-1.84260

C	-3.64290	3.07550	0.00620
C	-2.43660	3.20060	-0.68400
C	-1.81030	2.07520	-1.21730
C	-4.22520	1.81690	0.15490
C	-3.60470	0.68720	-0.37770
C	-2.39520	0.81660	-1.06390
O	-1.80830	-0.29340	-1.63930
H	-4.12780	3.95340	0.42030
H	-1.97910	4.17720	-0.80660
H	-0.87140	2.15590	-1.75110
H	-5.16560	1.71140	0.68630
H	-4.04820	-0.29610	-0.27080
C	4.50100	-0.52980	-1.00800
C	5.28920	0.61540	-0.89310
C	4.85460	1.66560	-0.08430
C	3.28970	-0.63550	-0.32660
C	2.86620	0.42190	0.48120
C	3.64490	1.57360	0.59970
H	4.82820	-1.35370	-1.63430
H	6.22980	0.68840	-1.42830
H	5.45530	2.56420	0.01380
H	2.67780	-1.52280	-0.42940
H	3.29310	2.38170	1.23190
F	0.51310	-1.33180	3.06740

33
6secondsubTSpyrazine.xyz

N	-1.24430	-1.83780	1.40630
C	-1.47910	-2.04460	0.15740
C	-0.76710	-1.38310	-0.90990
N	0.30930	-0.67240	-0.55980
C	0.53990	-0.45090	0.73260
C	-0.23160	-1.02830	1.71600
O	1.55150	0.39910	1.11150
F	-2.48750	-2.88470	-0.17530
F	-0.60450	-2.13860	-2.05130
C	-3.27570	2.98530	0.43980
C	-2.15230	3.07040	-0.38600
C	-1.70950	1.95850	-1.09810
C	-3.95730	1.77010	0.53790
C	-3.52090	0.65470	-0.17230
C	-2.38780	0.72330	-1.01200
O	-1.98290	-0.33960	-1.70880
H	-3.61940	3.85440	0.99180
H	-1.61620	4.01150	-0.47410

H	-0.84050	2.02320	-1.74490
H	-4.83530	1.69130	1.17330
H	-4.05250	-0.28960	-0.10400
C	4.60700	-0.63440	-0.69080
C	5.25960	0.59510	-0.78390
C	4.64980	1.73620	-0.26370
C	3.35530	-0.73290	-0.08590
C	2.75420	0.41770	0.42470
C	3.39530	1.65190	0.33700
H	5.07430	-1.52940	-1.08870
H	6.23400	0.66190	-1.25560
H	5.14650	2.69870	-0.32840
H	2.85300	-1.68940	-0.01530
H	2.90560	2.52940	0.74420
F	0.01070	-0.82130	3.03730

33
SecondSub2FirstINTpyrimidine.xyz

C	1.46470	-2.07600	-0.30740
N	1.38750	-2.09390	1.00700
C	0.30050	-1.53540	1.49560
N	-0.72270	-1.01370	0.85130
C	-0.63130	-1.01380	-0.47150
C	0.48460	-1.55040	-1.12360
O	-1.60430	-0.51000	-1.24260
F	0.18010	-1.57860	2.81880
C	3.30200	2.77400	-1.28000
C	2.04400	3.17840	-0.81430
C	1.45010	2.57010	0.28500
C	3.95210	1.73590	-0.59960
C	3.37300	1.11790	0.50170
C	2.08320	1.50170	1.01530
O	1.54520	0.94310	2.03120
H	1.51840	3.98560	-1.32270
H	0.47390	2.90030	0.63630
H	4.93200	1.40390	-0.93970
H	3.89580	0.31540	1.01890
C	-4.95330	-0.10420	0.23280
C	-5.01380	1.28440	0.35330
C	-3.92560	2.06590	-0.03370
C	-3.80760	-0.71700	-0.27260
C	-2.73450	0.08100	-0.64620
C	-2.77280	1.46420	-0.53730
H	-5.79890	-0.71490	0.52990
H	-5.90860	1.75600	0.74450

H	-3.96940	3.14590	0.05530
H	-3.74650	-1.79390	-0.37940
H	-1.91510	2.05190	-0.84370
H	3.76140	3.25410	-2.13870
F	2.55240	-2.61070	-0.86060
F	0.58690	-1.55660	-2.46050

32
SecondSub2PRODpyrimidine.xyz

C	-0.73030	2.32150	0.01540
N	-1.44660	1.21620	-0.00100
C	-0.74790	0.08790	-0.01820
N	0.57790	-0.03420	-0.02110
C	1.27430	1.09010	-0.00300
C	0.64710	2.34330	0.01580
O	2.61480	1.08310	-0.00470
C	-5.55930	-1.34650	-0.00430
C	-4.87280	-1.28720	-1.21700
C	-3.48330	-1.17190	-1.23280
C	-4.85440	-1.29480	1.19820
C	-3.46500	-1.17980	1.19380
C	-2.80060	-1.11710	-0.02450
O	-1.39740	-1.09300	-0.03600
H	-5.41640	-1.33230	-2.15440
H	-2.93260	-1.13050	-2.16560
H	-5.38370	-1.34590	2.14350
H	-2.90050	-1.14470	2.11860
C	4.33200	-1.93550	1.21430
C	4.81200	-2.46250	0.01520
C	4.53560	-1.81740	-1.18980
C	3.57290	-0.76640	1.21460
C	3.30530	-0.14440	0.00240
C	3.77570	-0.64810	-1.20230
H	4.54870	-2.43200	2.15390
H	5.40410	-3.37100	0.02070
H	4.91050	-2.22120	-2.12390
H	3.19730	-0.33980	2.13750
H	3.55350	-0.12930	-2.12770
H	-6.64000	-1.43800	0.00360
F	-1.39760	3.47550	0.03330
F	1.34800	3.48690	0.03430

33
SecondSub2SecondINTpyrimidine.xyz

C	1.49430	-2.03680	-0.39300
N	1.70110	-1.65510	0.82650
C	0.73790	-0.79800	1.38590
N	-0.43370	-0.47180	0.68230
C	-0.55520	-0.91410	-0.53680
C	0.40260	-1.70870	-1.17900
O	-1.66840	-0.61220	-1.28010
F	0.34400	-1.35160	2.64350
C	3.23640	2.96820	-0.84190
C	1.91080	3.18750	-0.46630
C	1.28200	2.32710	0.43310
C	3.93430	1.88640	-0.30380
C	3.31200	1.02440	0.59800
C	1.98030	1.23990	0.96670
O	1.37840	0.42690	1.89890
H	1.36230	4.03090	-0.87410
H	0.25320	2.48810	0.73270
H	4.96840	1.71260	-0.58490
H	3.84080	0.17980	1.02210
C	-4.87340	-0.04160	0.41910
C	-4.96050	1.34960	0.36450
C	-3.92640	2.08640	-0.21330
C	-3.75640	-0.69590	-0.09820
C	-2.73200	0.05130	-0.67110
C	-2.80610	1.43890	-0.73320
H	-5.67590	-0.62110	0.86340
H	-5.83160	1.85650	0.76580
H	-3.98950	3.16850	-0.26100
H	-3.67590	-1.77680	-0.06880
H	-1.99590	1.99680	-1.18900
H	3.72410	3.63920	-1.54150
F	2.44010	-2.84170	-0.94640
F	0.25830	-2.14570	-2.46840

33
SecondSub2TSpyrimidine.xyz

C	1.55800	-2.02800	-0.33310
N	1.59820	-1.87090	0.96130
C	0.59700	-1.11540	1.47840
N	-0.54270	-0.79040	0.81440
C	-0.53610	-0.98220	-0.48350
C	0.53510	-1.58810	-1.15160
O	-1.60440	-0.63790	-1.24950
F	0.36180	-1.39440	2.79100
C	3.29370	2.68240	-1.07070

C	2.00750	3.03470	-0.65270
C	1.35790	2.30330	0.33770
C	3.92580	1.59270	-0.46610
C	3.28650	0.86060	0.53080
C	1.97720	1.19150	0.95970
O	1.36360	0.51720	1.91950
H	1.50620	3.88650	-1.10520
H	0.36060	2.57960	0.66710
H	4.93080	1.31390	-0.77250
H	3.78330	0.02040	1.00440
C	-4.90290	-0.16130	0.30050
C	-4.96780	1.23080	0.35890
C	-3.89330	1.99580	-0.09470
C	-3.76680	-0.79140	-0.20590
C	-2.70420	-0.01390	-0.65080
C	-2.75330	1.37440	-0.60280
H	-5.73710	-0.76110	0.64870
H	-5.85390	1.71750	0.75200
H	-3.93970	3.07890	-0.05540
H	-3.70160	-1.87210	-0.26210
H	-1.91030	1.95270	-0.96330
H	3.79850	3.25400	-1.84310
F	2.57690	-2.70710	-0.89660
F	0.52980	-1.79740	-2.49280

32
SecondSub3PRODpyridazine.xyz

N	0.89770	2.71590	-0.30770
N	1.52000	1.52990	-0.17460
C	0.82760	0.45940	0.13720
C	-0.58200	0.45840	0.35710
C	-1.18980	1.68680	0.24010
C	-0.38230	2.78050	-0.10920
O	-1.17390	-0.69390	0.72570
C	5.54790	-1.20180	-0.24090
C	5.03710	-0.85520	1.01000
C	3.66650	-0.66720	1.18200
C	4.68500	-1.36570	-1.32430
C	3.31240	-1.17970	-1.16380
C	2.82530	-0.83060	0.08890
O	1.43520	-0.73680	0.27520
H	6.61460	-1.34910	-0.36960
H	5.70400	-0.73180	1.85640
H	3.25120	-0.40340	2.14790
H	5.07710	-1.64030	-2.29750

H	2.62580	-1.30800	-1.99270
C	-4.53860	-2.11490	0.74830
C	-4.95040	-1.90140	-0.56710
C	-4.09750	-1.25920	-1.46380
C	-3.28400	-1.68160	1.17240
C	-2.45250	-1.03860	0.26360
C	-2.83660	-0.82660	-1.05580
H	-5.19480	-2.61600	1.45140
H	-5.92870	-2.23710	-0.89270
H	-4.40690	-1.09680	-2.49060
H	-2.94550	-1.83510	2.19040
H	-2.16580	-0.34320	-1.75690
F	-2.49550	1.85900	0.44560
F	-0.96810	3.97560	-0.23720

33
SecondSub3TSpyridazine.xyz

N	-1.29410	2.10940	1.04680
N	-0.90450	1.07170	1.81500
C	-0.18500	0.07370	1.26050
C	0.43520	0.26050	-0.03500
C	0.02600	1.32750	-0.77030
C	-0.87910	2.22710	-0.16920
O	1.29330	-0.67980	-0.54880
F	0.63920	-0.54860	2.17160
C	-4.78560	-1.01340	-0.97850
C	-3.62930	-1.42170	-1.64780
C	-2.42000	-1.53390	-0.96690
C	-4.71510	-0.72470	0.38630
C	-3.50930	-0.83740	1.07410
C	-2.33230	-1.25110	0.41380
O	-1.18120	-1.39210	1.07250
H	-5.72790	-0.92930	-1.51050
H	-3.67080	-1.65330	-2.70870
H	-1.52050	-1.85590	-1.48300
H	-5.60820	-0.40930	0.91900
H	-3.45170	-0.62010	2.13550
C	4.82570	-1.53250	-0.71780
C	5.42460	-0.49410	-0.00190
C	4.62510	0.50530	0.54820
C	3.44480	-1.57100	-0.88270
C	2.65680	-0.56220	-0.32740
C	3.23860	0.47970	0.39140
H	5.43510	-2.31840	-1.15160
H	6.50120	-0.46610	0.12520

H	5.07680	1.31740	1.10850
H	2.96420	-2.37040	-1.43560
H	2.62520	1.25950	0.82570
F	0.49640	1.55800	-2.00680
F	-1.29740	3.29750	-0.88780

33
SecondSub5FirstINTpyridazine.xyz

N	-1.28230	-2.06750	1.26470
C	-0.80990	-2.12510	0.05730
C	0.10680	-1.20960	-0.48890
C	0.53540	-0.19880	0.33810
C	-0.01910	-0.20800	1.63260
N	-0.87120	-1.07780	2.08930
O	1.34130	0.81370	-0.08140
F	-1.22390	-3.13600	-0.70890
F	0.62470	-1.38990	-1.69450
C	-4.34370	2.22800	0.50260
C	-3.04440	2.61170	0.15080
C	-2.22920	1.78440	-0.61400
C	-4.80770	0.98170	0.06210
C	-4.00410	0.14230	-0.69830
C	-2.66890	0.50130	-1.08740
O	-1.92580	-0.27100	-1.79550
H	-4.97620	2.88010	1.09710
H	-2.66150	3.57560	0.48160
H	-1.22350	2.10040	-0.88190
H	-5.81470	0.66100	0.32330
H	-4.37760	-0.82380	-1.03260
C	4.82670	1.67800	-0.66550
C	5.49640	0.51040	-0.29430
C	4.76500	-0.58500	0.15710
C	3.43990	1.75020	-0.58700
C	2.72450	0.64420	-0.13240
C	3.37240	-0.52850	0.24180
H	5.38470	2.53860	-1.01860
H	6.57760	0.45710	-0.35660
H	5.27340	-1.49760	0.44930
H	2.90290	2.64830	-0.86980
H	2.81510	-1.38610	0.59930
F	0.36190	0.75710	2.47610

33
SecondSub5FirstINTpyrimidine.xyz

N	-1.12040	-2.02360	0.65030
C	-1.00600	-1.93240	-0.66070
C	0.05290	-1.31360	-1.29120
C	1.01740	-0.74830	-0.45920
N	0.91700	-0.82240	0.86640
C	-0.14260	-1.45210	1.31940
O	2.03530	-0.10270	-1.04730
F	-1.95410	-2.50800	-1.39110
F	0.13620	-1.23350	-2.62190
C	-4.63290	2.38570	1.31570
C	-3.39140	2.97200	1.03750
C	-2.51800	2.41320	0.11250
C	-4.97500	1.21490	0.62640
C	-4.11350	0.64430	-0.30200
C	-2.82710	1.20990	-0.61490
O	-2.02590	0.68390	-1.46310
H	-5.31060	2.82710	2.04020
H	-3.10060	3.88360	1.55790
H	-1.55670	2.88330	-0.08830
H	-5.93480	0.73860	0.82210
H	-4.39810	-0.26560	-0.82750
C	4.54890	2.07530	0.49610
C	5.44440	1.13730	1.00760
C	5.18460	-0.22590	0.86010
C	3.39030	1.65740	-0.15900
C	3.15160	0.29690	-0.29350
C	4.03370	-0.65550	0.20330
H	4.74770	3.13590	0.60520
H	6.34410	1.46620	1.51610
H	5.88060	-0.95950	1.25220
H	2.68280	2.36970	-0.56730
H	3.82560	-1.71120	0.07300
F	-0.24080	-1.52590	2.64990

32
SecondSub5PRODpyridazine.xyz

N	0.61700	3.04160	-0.26630
C	1.19370	1.90250	-0.51670
C	0.63670	0.62850	-0.27350
C	-0.63690	0.62880	0.27400
C	-1.19370	1.90300	0.51660
N	-0.61670	3.04190	0.26570
O	-1.22500	-0.53490	0.62270
F	2.41080	1.96260	-1.06490
C	5.09380	-1.57440	0.53610

C	4.57140	-1.98940	-0.68930
C	3.28560	-1.61380	-1.07050
C	4.31950	-0.78690	1.38600
C	3.02720	-0.40940	1.02170
C	2.53160	-0.82490	-0.20920
O	1.22450	-0.53550	-0.62160
H	6.09660	-1.86610	0.82770
H	5.16620	-2.60380	-1.35640
H	2.86310	-1.92130	-2.02000
H	4.71430	-0.46640	2.34400
H	2.41890	0.18560	1.69350
C	-4.57130	-1.99010	0.68870
C	-5.09350	-1.57490	-0.53680
C	-4.31920	-0.78670	-1.38620
C	-3.28580	-1.61420	1.07040
C	-2.53180	-0.82460	0.20960
C	-3.02720	-0.40880	-1.02120
H	-5.16610	-2.60500	1.35530
H	-6.09610	-1.86690	-0.82880
H	-4.71380	-0.46600	-2.34410
H	-2.86350	-1.92180	2.01990
H	-2.41890	0.18670	-1.69260
F	-2.41080	1.96360	1.06470

32
SecondSub5PRODpyrimidine.xyz

N	0.21370	3.27010	0.28660
C	1.09080	2.42550	-0.22510
C	0.81660	1.10430	-0.53700
C	-0.50120	0.69610	-0.25900
N	-1.40100	1.52660	0.25960
C	-0.97600	2.74920	0.49170
O	-0.81600	-0.57110	-0.53980
F	2.31080	2.90200	-0.45370
C	4.03890	-2.50370	0.88800
C	3.21440	-1.65070	1.61690
C	2.42160	-0.69950	0.97150
C	4.07120	-2.40360	-0.50430
C	3.28570	-1.46260	-1.16190
C	2.46550	-0.61690	-0.41700
O	1.72610	0.30040	-1.16110
H	4.65160	-3.23910	1.39750
H	3.18160	-1.71680	2.69910
H	1.78990	-0.03700	1.55110
H	4.70990	-3.06160	-1.08400

H	3.29680	-1.37130	-2.24210
C	-3.64810	-2.21090	1.13790
C	-4.62130	-2.13680	0.14160
C	-4.33070	-1.51590	-1.07310
C	-2.38340	-1.66290	0.92690
C	-2.11980	-1.04600	-0.28800
C	-3.06990	-0.96420	-1.29640
H	-3.86910	-2.69700	2.08180
H	-5.60280	-2.56620	0.30970
H	-5.08360	-1.46060	-1.85160
H	-1.61160	-1.71650	1.68600
H	-2.82280	-0.48370	-2.23600
F	-1.88140	3.57910	1.00870

33
SecondSub5SecondINTpyrimidine.xyz

N	-1.08070	-2.36400	0.77610
C	-1.13790	-1.80890	-0.40460
C	-0.41570	-0.64990	-0.87240
C	0.43030	-0.20660	0.21280
N	0.49670	-0.74930	1.41910
C	-0.26090	-1.79370	1.63750
O	1.20740	0.92030	-0.03460
F	-1.97230	-2.39740	-1.31250
F	0.38180	-0.96900	-2.09800
C	-4.21430	2.24600	0.72500
C	-2.94460	2.81660	0.63110
C	-1.94320	2.18880	-0.10860
C	-4.47970	1.04410	0.06850
C	-3.48360	0.41260	-0.67520
C	-2.21220	0.98670	-0.76840
O	-1.23900	0.39740	-1.54500
H	-4.99160	2.73560	1.30250
H	-2.73010	3.75220	1.13800
H	-0.95080	2.61830	-0.18480
H	-5.46550	0.59450	0.13400
H	-3.68090	-0.52020	-1.19070
C	4.69930	1.93850	0.17800
C	5.37540	0.73460	-0.02330
C	4.64120	-0.43270	-0.23140
C	3.30710	1.97430	0.18350
C	2.58140	0.79920	-0.02250
C	3.24740	-0.40950	-0.23540
H	5.25590	2.85620	0.33920
H	6.45970	0.70710	-0.02030

H	5.15410	-1.37480	-0.39750
H	2.76990	2.90240	0.34670
H	2.68120	-1.31470	-0.41440
F	-0.20610	-2.35090	2.88080

33
SecondSub5TSpyridazine.xyz

N	-1.24560	-2.13560	1.36460
C	-0.94550	-2.11890	0.11100
C	-0.18930	-1.11550	-0.57110
C	0.40970	-0.20710	0.31040
C	0.02780	-0.28070	1.64810
N	-0.75460	-1.17630	2.19380
O	1.21540	0.80600	-0.16000
F	-1.43280	-3.12360	-0.64480
F	0.47760	-1.46650	-1.69470
C	-4.34760	2.06940	0.36410
C	-3.04330	2.53380	0.18110
C	-2.09790	1.75970	-0.48780
C	-4.69420	0.80970	-0.13440
C	-3.75480	0.02630	-0.79630
C	-2.42920	0.48320	-1.00770
O	-1.55180	-0.25420	-1.66070
H	-5.08190	2.67800	0.88240
H	-2.75990	3.51140	0.56270
H	-1.08810	2.12860	-0.63710
H	-5.70520	0.43430	0.00140
H	-4.02200	-0.95330	-1.18310
C	4.72170	1.66630	-0.61280
C	5.37350	0.48130	-0.26540
C	4.61980	-0.62220	0.12730
C	3.33370	1.74800	-0.56610
C	2.59130	0.63480	-0.16990
C	3.22650	-0.55570	0.17670
H	5.29540	2.53410	-0.92090
H	6.45570	0.42070	-0.30140
H	5.11290	-1.55020	0.39780
H	2.81340	2.66170	-0.83090
H	2.64800	-1.42030	0.47820
F	0.53420	0.64010	2.49110

33
SecondSub6FirstINTpyrimidine.xyz

C	-0.58740	-1.24180	1.76780

N	-1.69030	-1.80850	1.33210
C	-1.71650	-2.02070	0.02860
C	-0.66540	-1.69860	-0.80860
C	0.44940	-1.11570	-0.20870
N	0.48980	-0.88940	1.10350
O	1.48980	-0.81620	-1.00150
F	-2.76830	-2.66360	-0.45950
C	-1.22780	3.72380	0.02370
C	-0.87730	2.97520	-1.10740
C	-1.50590	1.77040	-1.40040
C	-2.23900	3.22540	0.85620
C	-2.87830	2.02460	0.57610
C	-2.55250	1.22610	-0.57630
O	-3.13890	0.12050	-0.84200
H	-0.09740	3.34240	-1.77270
H	-1.22120	1.20240	-2.28390
H	-2.53010	3.78850	1.74160
H	-3.66340	1.65240	1.23200
C	4.97320	-0.30220	0.02200
C	4.95560	1.04430	0.38240
C	3.76560	1.76970	0.30830
C	3.80310	-0.92890	-0.40720
C	2.63060	-0.18830	-0.47120
C	2.59090	1.15660	-0.12360
H	5.89590	-0.86990	0.07370
H	5.86660	1.52930	0.71560
H	3.74900	2.81860	0.58400
H	3.79490	-1.97360	-0.69620
H	1.66060	1.70890	-0.19430
H	-0.73520	4.66560	0.24520
F	-0.71620	-1.92050	-2.12910
F	-0.55010	-1.00260	3.08310

32
SecondSub6PRODpyridazine.xyz

N	0.27360	-1.47860	1.12870
N	1.36850	-0.85320	0.66060
C	1.25430	0.34890	0.14130
C	0.01610	1.02170	0.04210
C	-1.11030	0.39430	0.51540
C	-0.88000	-0.88950	1.06480
O	-2.29890	1.03700	0.52520
C	6.14590	-0.66630	-0.31430
C	5.29120	-0.89250	-1.39310
C	4.00240	-0.36070	-1.39200

C	5.71320	0.09780	0.76930
C	4.42620	0.63420	0.78150
C	3.59060	0.39310	-0.30080
O	2.32700	1.01080	-0.33510
H	5.62560	-1.48170	-2.24010
H	3.32650	-0.52070	-2.22420
H	6.37650	0.28040	1.60770
H	4.07370	1.23490	1.61210
C	-5.83380	0.26960	0.05090
C	-5.79710	-0.63390	-1.01180
C	-4.57170	-0.99940	-1.56560
C	-4.65350	0.80020	0.56540
C	-3.44150	0.41940	0.00030
C	-3.38160	-0.47020	-1.06640
H	-6.78300	0.56100	0.48720
H	-6.71810	-1.04770	-1.40720
H	-4.53390	-1.69510	-2.39680
H	-4.65940	1.49930	1.39350
H	-2.43100	-0.74010	-1.51240
H	7.14850	-1.07990	-0.32050
F	-0.04720	2.23740	-0.49470
F	-1.92720	-1.55630	1.56500

32
SecondSub6PRODpyrimidine.xyz

C	0.00000	-1.40640	-0.00060
N	-1.18900	-0.84870	-0.00040
C	-1.18840	0.48440	0.00010
C	0.00000	1.20940	0.00040
C	1.18840	0.48440	0.00010
N	1.18900	-0.84870	-0.00040
O	2.33580	1.17810	0.00040
C	-6.06280	-0.69370	-0.00020
C	-5.43350	-0.39360	1.20780
C	-4.17430	0.20530	1.21420
C	-5.43400	-0.39180	-1.20790
C	-4.17490	0.20700	-1.21400
C	-3.56550	0.49280	0.00020
O	-2.33580	1.17810	0.00040
H	-5.92100	-0.62310	2.14890
H	-3.67240	0.45170	2.14280
H	-5.92190	-0.62000	-2.14920
H	-3.67320	0.45480	-2.14240
C	5.43400	-0.39180	-1.20790
C	6.06280	-0.69370	-0.00020

C	5.43350	-0.39360	1.20780
C	4.17490	0.20700	-1.21400
C	3.56550	0.49280	0.00020
C	4.17430	0.20530	1.21420
H	5.92190	-0.62000	-2.14920
H	7.04310	-1.15750	-0.00030
H	5.92100	-0.62310	2.14890
H	3.67320	0.45480	-2.14240
H	3.67240	0.45170	2.14280
H	-7.04310	-1.15750	-0.00030
F	-0.00000	2.55210	0.00100
F	0.00000	-2.74350	-0.00120

33
SecondSub6SecondINTpyrimidine.xyz

C	-0.62820	-1.13120	1.74330
N	-1.82840	-1.28400	1.26890
C	-1.96040	-1.33970	-0.11880
C	-0.68310	-1.37990	-0.83730
C	0.48050	-1.17150	-0.17580
N	0.55330	-1.04720	1.17270
O	1.65190	-1.14440	-0.89710
F	-2.77040	-2.45520	-0.47690
C	-1.50070	3.64360	-0.11070
C	-1.07910	2.89660	-1.21110
C	-1.52250	1.58620	-1.38390
C	-2.37690	3.07460	0.81400
C	-2.81910	1.76240	0.64970
C	-2.39050	1.01460	-0.44900
O	-2.85090	-0.27250	-0.62410
H	-0.40330	3.33360	-1.93940
H	-1.20080	0.99720	-2.23480
H	-2.71350	3.65070	1.67010
H	-3.49060	1.30290	1.36540
C	5.08790	-0.25420	-0.03590
C	4.95700	1.08610	0.32430
C	3.69720	1.68700	0.29530
C	3.96750	-0.99380	-0.41250
C	2.71630	-0.38450	-0.43450
C	2.57230	0.95870	-0.08610
H	6.06220	-0.73160	-0.01890
H	5.82820	1.65970	0.62200
H	3.58690	2.73130	0.56890
H	4.05000	-2.03860	-0.69060
H	1.59370	1.42390	-0.11370

H	-1.15460	4.66340	0.02140
F	-0.72820	-1.47420	-2.20380
F	-0.57740	-1.04370	3.10410

33
SecondSub6TSpyridazine.xyz

N	1.08090	-1.32100	-1.96130
N	2.12030	-1.58120	-1.13260
C	1.91260	-1.64890	0.19520
C	0.55860	-1.69580	0.69600
C	-0.48060	-1.46320	-0.14650
C	-0.12180	-1.26230	-1.50770
O	-1.78100	-1.55990	0.27560
F	2.75190	-2.55490	0.82000
C	1.89170	3.55530	-0.34740
C	1.27180	3.09160	0.81550
C	1.50330	1.79580	1.27100
C	2.75720	2.70650	-1.04050
C	3.00390	1.41360	-0.58390
C	2.38260	0.92950	0.58700
O	2.63110	-0.29460	1.06070
H	0.59990	3.74220	1.36840
H	1.02470	1.43170	2.17520
H	3.24990	3.05710	-1.94320
H	3.68480	0.75800	-1.11400
C	-4.87550	0.35710	0.26020
C	-4.41410	1.67110	0.16410
C	-3.04360	1.91270	0.09600
C	-3.97690	-0.70540	0.28120
C	-2.60890	-0.44650	0.21010
C	-2.13030	0.85790	0.12090
H	-5.94000	0.15510	0.31470
H	-5.11660	2.49710	0.14460
H	-2.67210	2.92970	0.02670
H	-4.31730	-1.73250	0.35100
H	-1.06510	1.05550	0.08610
H	1.71010	4.56480	-0.70200
F	0.35360	-1.94120	2.00160
F	-1.12180	-1.02830	-2.39570

33
SecondSum5SecondINTpyridazine.xyz

N	-1.19930	-2.15780	1.43920
C	-1.11520	-1.96820	0.17500

C	-0.37930	-0.93360	-0.57300
C	0.36270	-0.15870	0.40180
C	0.20400	-0.41980	1.74220
N	-0.52860	-1.35710	2.31420
O	1.13840	0.90740	-0.04860
F	-1.82690	-2.82710	-0.61670
F	0.48140	-1.54550	-1.55460
C	-4.38230	2.06370	0.02540
C	-3.09860	2.60890	-0.01570
C	-2.03020	1.86060	-0.50820
C	-4.59530	0.76340	-0.43220
C	-3.53140	0.00860	-0.92450
C	-2.24950	0.55990	-0.96550
O	-1.21240	-0.17220	-1.51230
H	-5.21140	2.64950	0.40830
H	-2.92620	3.62090	0.33670
H	-1.02770	2.27060	-0.54400
H	-5.59110	0.33270	-0.40530
H	-3.68060	-1.00450	-1.28030
C	4.60920	1.82540	-0.64480
C	5.28820	0.63040	-0.39930
C	4.56150	-0.50140	-0.03480
C	3.22440	1.88900	-0.52220
C	2.50590	0.74880	-0.15310
C	3.17290	-0.45230	0.08930
H	5.15920	2.71610	-0.93100
H	6.36790	0.58350	-0.49210
H	5.07470	-1.43880	0.15550
H	2.68620	2.81280	-0.70550
H	2.61280	-1.33760	0.36110
F	0.88890	0.36560	2.61350

33
SecondSum6FirstINTpyridazine.xyz

N	0.92760	-1.45020	-2.04100
N	1.96080	-1.91360	-1.30940
C	1.78040	-2.12870	-0.03870
C	0.56740	-1.92460	0.63580
C	-0.50790	-1.48560	-0.10160
C	-0.22340	-1.26430	-1.47010
O	-1.73290	-1.41630	0.46650
F	2.79990	-2.64920	0.63850
C	1.81900	3.80890	-0.47250
C	1.10530	3.43670	0.67320
C	1.32000	2.20960	1.29080

C	2.76970	2.91140	-0.97610
C	2.99930	1.68370	-0.36790
C	2.28320	1.25800	0.80530
O	2.48330	0.12570	1.37010
H	0.36560	4.11830	1.09010
H	0.75920	1.93840	2.18340
H	3.34150	3.17890	-1.86350
H	3.74720	1.00420	-0.77120
C	-4.82800	0.48690	0.15840
C	-4.35700	1.79520	0.04280
C	-2.98430	2.03790	0.05000
C	-3.93360	-0.57450	0.27240
C	-2.56840	-0.30900	0.27470
C	-2.07550	0.98630	0.17100
H	-5.89420	0.28770	0.15600
H	-5.05540	2.61980	-0.04810
H	-2.60880	3.05240	-0.03050
H	-4.27750	-1.59860	0.36030
H	-1.00900	1.18410	0.20700
H	1.64650	4.76770	-0.95180
F	0.45040	-2.20190	1.92800
F	-1.21810	-0.83900	-2.25930

33

SecondSum6SecondINTpyridazine.xyz

N	0.99810	-1.37400	-2.02000
N	2.05680	-1.30500	-1.19030
C	1.87980	-1.34210	0.18160
C	0.49650	-1.36780	0.66330
C	-0.54740	-1.41300	-0.19800
C	-0.20940	-1.42200	-1.57630
O	-1.84860	-1.52070	0.23840
F	2.56680	-2.51440	0.75380
C	1.98920	3.68670	-0.02000
C	1.37410	3.09430	1.08300
C	1.59090	1.74630	1.36840
C	2.82910	2.92440	-0.83270
C	3.05140	1.57720	-0.55170
C	2.43120	0.98550	0.55190
O	2.68600	-0.32920	0.87910
H	0.72280	3.68030	1.72380
H	1.11850	1.27380	2.22260
H	3.31450	3.37960	-1.69040
H	3.69580	0.97100	-1.17500
C	-4.92550	0.41840	0.36000

C	-4.46900	1.72230	0.15740
C	-3.10740	1.94790	-0.03120
C	-4.03220	-0.64820	0.37080
C	-2.67100	-0.40720	0.18070
C	-2.19890	0.88840	-0.02020
H	-5.98340	0.22760	0.50780
H	-5.16790	2.55160	0.14790
H	-2.73830	2.95640	-0.18620
H	-4.37030	-1.66680	0.52490
H	-1.14010	1.07480	-0.15440
H	1.82020	4.73550	-0.24110
F	0.28550	-1.33670	2.00130
F	-1.22860	-1.46110	-2.48450

33
SecondSub3SecondINTpyridazine.xyz

N	-1.31090	2.17660	1.01980
N	-1.16600	1.03830	1.71030
C	-0.38390	0.00330	1.20650
C	0.34860	0.26090	-0.04470
C	0.13750	1.42780	-0.70070
C	-0.73080	2.37160	-0.11660
O	1.16590	-0.72520	-0.56470
F	0.58150	-0.40660	2.21110
C	-4.67430	-1.40620	-1.03540
C	-3.46320	-1.74810	-1.63750
C	-2.27410	-1.68590	-0.91130
C	-4.69020	-1.00590	0.30120
C	-3.50500	-0.94180	1.03190
C	-2.29180	-1.28440	0.42750
O	-1.12670	-1.28110	1.15900
H	-5.59870	-1.45600	-1.60160
H	-3.44160	-2.06390	-2.67580
H	-1.32700	-1.95170	-1.36800
H	-5.62870	-0.74040	0.77790
H	-3.50130	-0.62850	2.06820
C	4.67560	-1.63780	-0.87820
C	5.31600	-0.65090	-0.12670
C	4.55150	0.33280	0.49740
C	3.28990	-1.63890	-1.00790
C	2.53530	-0.64650	-0.37930
C	3.16160	0.34280	0.37790
H	5.25610	-2.41190	-1.36950
H	6.39620	-0.65080	-0.02840
H	5.03480	1.10390	1.08880

H	2.77960	-2.39820	-1.59030
H	2.57390	1.10090	0.87890
F	0.76370	1.70460	-1.86290
F	-0.95250	3.55140	-0.76600

33
SecondSub5TSpyrimidine.xyz

N	-1.12890	-2.31710	0.72330
C	-1.07940	-1.88390	-0.51080
C	-0.37030	-0.73890	-0.98720
C	0.46510	-0.24660	0.06520
N	0.42640	-0.65950	1.32550
C	-0.37400	-1.66300	1.58360
O	1.28800	0.81910	-0.25180
F	-1.81310	-2.56870	-1.42670
F	0.27400	-0.91280	-2.22720
C	-4.25110	2.02390	0.96520
C	-3.02240	2.66340	0.78790
C	-2.06220	2.12450	-0.06540
C	-4.51040	0.83830	0.27530
C	-3.55500	0.29620	-0.58200
C	-2.31790	0.93770	-0.77590
O	-1.40410	0.43690	-1.62870
H	-4.99760	2.44550	1.63060
H	-2.80930	3.58520	1.32140
H	-1.10420	2.61450	-0.20640
H	-5.46230	0.33180	0.40630
H	-3.75330	-0.62240	-1.12490
C	4.76080	1.82850	0.18570
C	5.43340	0.60670	0.14380
C	4.70340	-0.56990	-0.02140
C	3.37360	1.87430	0.07200
C	2.65360	0.69040	-0.09240
C	3.31490	-0.53740	-0.14190
H	5.31540	2.75240	0.31310
H	6.51350	0.57230	0.23720
H	5.21520	-1.52610	-0.06200
H	2.83710	2.81610	0.10670
H	2.75250	-1.45270	-0.28110
F	-0.42010	-2.09560	2.87070

10
Tetrafluoropyridizine.xyz

C	-0.98089	0.02269	0.00000

C	-0.54510	-1.31314	0.00000
N	0.70379	-1.68036	0.00000
N	1.66371	-0.74249	0.00000
C	1.32542	0.51455	0.00000
C	0.00000	0.98105	0.00000
F	2.30495	1.41339	0.00000
F	-0.27864	2.27469	0.00000
F	-2.26774	0.33130	0.00000
F	-1.46624	-2.27172	0.00000

22
TFP2subPhenoxy.xyz

C	1.43420	-0.50830	-1.16490
N	1.40960	0.79320	-1.19850
C	1.25910	1.40200	0.00020
N	1.40920	0.79300	1.19880
C	1.43380	-0.50860	1.16500
C	1.42870	-1.26440	-0.00010
F	1.51410	-1.15380	2.33940
F	1.47180	-2.61490	-0.00020
F	1.51500	-1.15330	-2.33950
F	1.68110	2.68510	0.00040
C	-3.47790	-1.00850	-0.00010
C	-2.98570	-0.49600	1.20360
C	-2.01400	0.50030	1.20840
C	-2.98590	-0.49580	-1.20390
C	-2.01410	0.50050	-1.20860
C	-1.49340	1.03140	-0.00010
H	-4.23830	-1.78310	-0.00020
H	-3.36480	-0.87850	2.14810
H	-1.63520	0.90020	2.14440
H	-3.36510	-0.87810	-2.14840
H	-1.63550	0.90050	-2.14460
O	-0.57980	1.98150	-0.00000

22
TFP2subPhenoxy2ndInt.xyz

C	1.44890	-0.56370	-1.16270
N	0.96820	0.63320	-1.22390
C	0.72650	1.28980	0.00020
N	0.96800	0.63290	1.22420
C	1.44870	-0.56400	1.16280
C	1.74030	-1.26860	-0.00000
F	1.67860	-1.19390	2.34220

F	2.20840	-2.55160	-0.00020
F	1.67890	-1.19330	-2.34230
F	1.50200	2.49370	0.00050
C	-3.89450	-0.67250	-0.00040
C	-3.33720	-0.24690	1.20570
C	-2.22470	0.59330	1.20920
C	-3.33700	-0.24660	-1.20630
C	-2.22450	0.59370	-1.20940
C	-1.66500	1.01250	0.00000
H	-4.76110	-1.32560	-0.00060
H	-3.76880	-0.56930	2.14800
H	-1.77850	0.92930	2.13730
H	-3.76840	-0.56870	-2.14880
H	-1.77810	0.92990	-2.13730
O	-0.59950	1.88870	0.00020

22

TFP2subPhenoxyFirstInt.xyz

C	1.44560	-0.47750	-1.16860
N	1.61230	0.82370	-1.18710
C	1.65000	1.39730	0.00030
N	1.61190	0.82350	1.18740
C	1.44510	-0.47780	1.16870
C	1.34690	-1.21850	-0.00000
F	1.38380	-1.10720	2.33740
F	1.18050	-2.54490	-0.00020
F	1.38460	-1.10670	-2.33740
F	1.86170	2.70530	0.00040
C	-3.62560	-1.16740	-0.00010
C	-3.16640	-0.61140	1.20090
C	-2.27760	0.45660	1.20960
C	-3.16660	-0.61120	-1.20120
C	-2.27780	0.45680	-1.20980
C	-1.77990	1.05650	-0.00010
H	-4.32100	-2.00120	-0.00020
H	-3.51070	-1.02350	2.14830
H	-1.93330	0.87810	2.15230
H	-3.51110	-1.02320	-2.14860
H	-1.93360	0.87850	-2.15250
O	-0.95860	2.03900	-0.00010

22

TFP4subPhenoxy.xyz

C	-1.38510	0.42810	1.29730

C	-1.53180	0.91180	0.02940
C	-1.42290	-0.02840	-1.02460
N	-1.36570	-1.33440	-0.74190
C	-1.23580	-1.63680	0.52720
N	-1.23790	-0.86300	1.59020
F	-1.11610	-2.95060	0.78730
F	-1.40600	1.27510	2.33610
F	-1.67210	2.22650	-0.22990
F	-1.99980	0.28650	-2.19780
C	3.60180	0.01330	0.77090
C	3.26950	-1.00300	-0.13040
C	2.18350	-0.86640	-0.98880
C	2.82670	1.17550	0.79200
C	1.74020	1.32310	-0.06580
C	1.38370	0.30820	-0.99500
H	4.45040	-0.09700	1.43870
H	3.86370	-1.91310	-0.16010
H	1.92840	-1.65610	-1.68990
H	3.07280	1.97590	1.48550
H	1.14590	2.23240	-0.05170
O	0.36620	0.44380	-1.81190

22
TFP4subPhenoxy1stIntermediate.xyz

C	-1.23850	0.42590	1.25270
C	-1.56070	0.87810	-0.00980
C	-1.67460	-0.10810	-0.98450
N	-1.52510	-1.39000	-0.72120
C	-1.21640	-1.66850	0.52840
N	-1.06940	-0.85160	1.54710
F	-1.04510	-2.96300	0.80030
F	-1.10170	1.30060	2.24610
F	-1.72420	2.17480	-0.28450
F	-2.07850	0.22250	-2.19980
C	3.70420	-0.02540	0.90540
C	3.40520	-0.99590	-0.06010
C	2.38960	-0.80110	-0.98760
C	2.95120	1.15430	0.91160
C	1.93070	1.36390	-0.00940
C	1.59830	0.39870	-1.02100
H	4.50090	-0.18280	1.62590
H	3.97740	-1.92180	-0.08470
H	2.17160	-1.56260	-1.73370
H	3.16470	1.92330	1.65210
H	1.35880	2.28950	0.00540

O	0.66270	0.58880	-1.87760

22
TFP4subPhenoxy2ndIntermediate.xyz

C	-1.54810	0.39760	1.31770
C	-1.29380	0.90940	0.09590
C	-0.93450	0.00870	-1.00830
N	-0.94730	-1.34330	-0.65800
C	-1.23950	-1.64660	0.57060
N	-1.54200	-0.91060	1.62200
F	-1.22560	-2.98260	0.83300
F	-1.83370	1.23050	2.34560
F	-1.29550	2.25540	-0.14250
F	-1.82440	0.22170	-2.10950
C	3.86810	0.11630	0.48380
C	3.44450	-0.94890	-0.31160
C	2.24420	-0.86710	-1.01610
C	3.08750	1.26950	0.56600
C	1.88730	1.35850	-0.13760
C	1.46500	0.28790	-0.92920
H	4.80240	0.05010	1.03160
H	4.04820	-1.84800	-0.38330
H	1.89970	-1.68660	-1.63550
H	3.41240	2.10420	1.17900
H	1.27320	2.25000	-0.08370
O	0.30800	0.39320	-1.67470

22
TFP5subPhenoxy1stINT.xyz

N	1.48860	1.12310	1.09670
C	1.62310	-0.18320	1.19030
C	1.70520	-1.02150	0.09210
C	1.60320	-0.38620	-1.13400
N	1.46640	0.91600	-1.26570
C	1.42380	1.58600	-0.13320
F	1.29740	2.90670	-0.24770
F	1.67230	-0.70660	2.40930
F	1.86460	-2.33640	0.20550
F	1.64000	-1.11150	-2.24460
C	-3.90970	1.26910	-0.06250
C	-3.47430	0.62960	-1.23040
C	-2.49130	-0.35140	-1.19320
C	-3.32770	0.88690	1.15290
C	-2.34440	-0.09360	1.20720

C	-1.86560	-0.77210	0.03220
H	-4.67800	2.03550	-0.09780
H	-3.91130	0.90700	-2.18850
H	-2.16340	-0.83660	-2.11090
H	-3.64870	1.36780	2.07570
H	-1.90310	-0.37690	2.16110
O	-0.95400	-1.67130	0.07150

22
TFP5subPhenoxy2ndINT.xyz

N	1.65940	1.20940	1.07130
C	1.16860	-0.00220	1.15390
C	0.86740	-0.90660	0.07440
C	1.14770	-0.18950	-1.14320
N	1.63810	1.01870	-1.26670
C	1.87420	1.66160	-0.14420
F	2.37110	2.92440	-0.25190
F	0.92180	-0.47170	2.41350
F	1.68680	-2.17970	0.17180
F	0.87840	-0.85640	-2.30480
C	-3.88350	0.73230	-0.05470
C	-3.33250	0.19420	-1.21790
C	-2.16590	-0.56680	-1.15720
C	-3.26370	0.50100	1.17350
C	-2.09820	-0.26090	1.24150
C	-1.55010	-0.79850	0.07460
H	-4.79110	1.32470	-0.10460
H	-3.80980	0.36810	-2.17700
H	-1.72420	-0.98720	-2.05320
H	-3.68770	0.91410	2.08320
H	-1.60780	-0.44800	2.18970
O	-0.43110	-1.60500	0.14040

22
TFP5subPhenoxyTS.xyz

N	1.58660	1.18090	1.07830
C	1.34990	-0.10720	1.15830
C	1.10080	-0.99840	0.07300
C	1.34930	-0.28960	-1.14010
N	1.58370	0.99490	-1.26410
C	1.68240	1.66810	-0.13870
F	1.92530	2.99800	-0.24480
F	1.26850	-0.63510	2.40730
F	1.71150	-2.26300	0.17320

F	1.26800	-1.00720	-2.28990
C	-3.65410	0.96340	-0.04980
C	-3.16380	0.37210	-1.21600
C	-2.08060	-0.50210	-1.16240
C	-3.04940	0.66810	1.17330
C	-1.96780	-0.20820	1.23360
C	-1.47270	-0.81760	0.06630
H	-4.49780	1.64450	-0.09370
H	-3.62540	0.59680	-2.17310
H	-1.69330	-0.96470	-2.06480
H	-3.42190	1.12380	2.08620
H	-1.49660	-0.44460	2.18220
O	-0.44760	-1.68900	0.12090

21
TFPprod2.xyz

C	2.61460	-0.90190	0.00020
N	1.45530	-1.50970	0.00000
C	0.38460	-0.71410	-0.00040
N	0.38890	0.61550	-0.00080
C	1.57090	1.18650	-0.00050
C	2.76230	0.47950	0.00000
F	1.60970	2.51460	-0.00080
F	3.95820	1.07630	0.00030
F	3.70570	-1.66050	0.00070
C	-4.45600	0.59740	0.00060
C	-3.83630	0.27800	-1.20740
C	-2.59470	-0.35640	-1.21400
C	-3.83520	0.27890	1.20830
C	-2.59360	-0.35540	1.21410
C	-1.99250	-0.65930	-0.00010
H	-5.42260	1.08910	0.00090
H	-4.31800	0.51990	-2.14850
H	-2.10120	-0.61840	-2.14280
H	-4.31600	0.52160	2.14960
H	-2.09910	-0.61660	2.14280
O	-0.78260	-1.37610	-0.00040

21
TFPprod4.xyz

C	-1.59790	1.60110	-0.00000
N	-0.46310	0.93590	-0.00000
C	-0.57000	-0.38890	-0.00000
C	-1.82400	-1.01360	0.00000

C	-2.91970	-0.17530	0.00000
N	-2.83010	1.14180	-0.00000
F	-1.48210	2.92810	-0.00000
F	-1.94600	-2.34530	0.00000
F	-4.14010	-0.70020	0.00000
C	4.38160	0.36620	0.00000
C	3.72970	0.11970	-1.20810
C	2.42420	-0.36980	-1.21490
C	3.72970	0.11980	1.20810
C	2.42420	-0.36970	1.21490
C	1.79520	-0.60230	0.00000
H	5.39780	0.74500	0.00000
H	4.23520	0.30610	-2.14920
H	1.90290	-0.57310	-2.14310
H	4.23520	0.30620	2.14920
H	1.90280	-0.57300	2.14310
O	0.50680	-1.17780	-0.00000

21
TFPprod5.xyz

N	-2.39090	-1.31150	0.15610
C	-1.17550	-1.00040	0.55110
C	-0.64010	0.28610	0.49970
C	-1.52240	1.23580	-0.01150
N	-2.74850	0.96580	-0.40840
C	-3.09920	-0.29800	-0.29860
F	-4.33080	-0.59110	-0.69820
F	-0.42370	-1.98640	1.02100
F	-1.12570	2.49650	-0.10160
C	4.16170	-0.38740	-0.88620
C	4.13580	0.11370	0.41650
C	2.92600	0.43450	1.02400
C	2.96630	-0.56090	-1.57870
C	1.74420	-0.24020	-0.98440
C	1.74060	0.25360	0.31570
H	5.10600	-0.63860	-1.35610
H	5.06030	0.25390	0.96610
H	2.88510	0.82180	2.03550
H	2.97380	-0.94550	-2.59290
H	0.82240	-0.36740	-1.53940
O	0.57820	0.62560	0.99710

35
TFPsecondSub2TS.xyz

C	-0.11150	2.50510	1.21670
C	-0.05610	2.70820	-0.12690
C	-0.75300	1.77800	-0.96740
N	-1.56240	0.87990	-0.35510
C	-1.51590	0.80730	0.95850
N	-0.82970	1.56010	1.81510
O	-2.28230	-0.13420	1.58910
F	0.59170	3.31730	2.03150
F	0.71640	3.67870	-0.68480
F	-1.32400	2.34310	-2.08560
C	3.41410	-1.26690	-0.12360
C	2.36520	-1.93670	-0.76390
C	1.34860	-1.20140	-1.34290
C	3.45260	0.12980	-0.07200
C	2.43670	0.86120	-0.66050
C	1.35850	0.21480	-1.31500
O	0.39710	0.88980	-1.92000
N	4.48280	-2.03380	0.48790
O	5.39540	-1.42690	1.05270
O	4.43750	-3.26410	0.42030
H	2.35840	-3.01810	-0.79080
H	0.52440	-1.69780	-1.84210
H	4.28080	0.62360	0.41850
H	2.47140	1.94340	-0.65080
C	-5.17540	-1.61640	-0.12320
C	-4.65460	-2.85500	-0.49750
C	-3.32920	-3.16950	-0.19650
C	-4.37580	-0.69050	0.54540
C	-3.05470	-1.01590	0.83430
C	-2.52300	-2.24920	0.47200
H	-6.20690	-1.36790	-0.35020
H	-5.27990	-3.57390	-1.01600
H	-2.91980	-4.13330	-0.48020
H	-4.76710	0.27480	0.84660
H	-1.49270	-2.48040	0.71800

35

TFPsecSub2firstINT.xyz

C	-0.14630	2.41310	1.18200
C	-0.35580	2.81710	-0.12580
C	-1.31400	2.08820	-0.81210
N	-1.97760	1.08910	-0.27920
C	-1.67440	0.79430	0.98090
N	-0.78800	1.42160	1.75230
O	-2.30100	-0.22700	1.59420

F	0.75940	3.05720	1.91580
F	0.33070	3.81900	-0.68690
F	-1.61960	2.43180	-2.05530
C	3.56730	-1.43590	-0.09870
C	2.54070	-2.09850	-0.80930
C	1.60710	-1.37800	-1.50710
C	3.62470	-0.02400	-0.10860
C	2.69270	0.70160	-0.80360
C	1.62350	0.07270	-1.55760
O	0.77240	0.73600	-2.20450
N	4.53330	-2.18210	0.61660
O	5.43940	-1.58130	1.22820
O	4.46140	-3.42750	0.62230
H	2.50940	-3.18090	-0.79130
H	0.81870	-1.88430	-2.05640
H	4.41610	0.46890	0.44250
H	2.73550	1.78690	-0.81210
C	-5.29190	-1.68710	0.01890
C	-4.76430	-2.88080	-0.47420
C	-3.41230	-3.17150	-0.29460
C	-4.47320	-0.77930	0.68920
C	-3.12790	-1.08480	0.85060
C	-2.58360	-2.26960	0.37150
H	-6.34390	-1.45960	-0.11560
H	-5.40660	-3.58430	-0.99280
H	-2.99830	-4.10040	-0.67170
H	-4.86810	0.15050	1.08230
H	-1.53070	-2.47840	0.52350

35
TFPsecSub2secondINT.xyz

C	-0.20390	2.48910	1.20850
C	-0.03410	2.60250	-0.12850
C	-0.64100	1.60610	-1.00540
N	-1.44440	0.68400	-0.35440
C	-1.53290	0.73820	0.95170
N	-0.95610	1.57040	1.81750
O	-2.33130	-0.18680	1.58730
F	0.42210	3.36730	2.02780
F	0.77370	3.55980	-0.68220
F	-1.36980	2.23740	-2.05210
C	3.37280	-1.32010	-0.15810
C	2.30430	-1.96890	-0.77690
C	1.28920	-1.20620	-1.33470
C	3.45110	0.07090	-0.10350

C	2.43620	0.82620	-0.67070
C	1.34430	0.19380	-1.28630
O	0.36300	0.91410	-1.88840
N	4.44040	-2.11730	0.44620
O	5.36190	-1.52710	1.00530
O	4.36690	-3.34150	0.37000
H	2.27290	-3.04940	-0.80630
H	0.44240	-1.67940	-1.81620
H	4.29860	0.54490	0.37220
H	2.48720	1.90690	-0.65650
C	-5.19610	-1.68090	-0.15530
C	-4.67900	-2.93040	-0.49710
C	-3.36200	-3.24920	-0.16450
C	-4.40050	-0.74990	0.51090
C	-3.08690	-1.07820	0.83350
C	-2.56110	-2.32360	0.50310
H	-6.22120	-1.42820	-0.40580
H	-5.30020	-3.65400	-1.01420
H	-2.95510	-4.22130	-0.42330
H	-4.78930	0.22400	0.78740
H	-1.53760	-2.55890	0.77240

10
TFPyrazine.xyz

N	1.37491	-0.00000	0.00000
C	0.69691	-1.12114	0.00000
C	-0.69691	-1.12114	0.00000
N	-1.37491	0.00000	0.00000
C	-0.69691	1.12114	0.00000
C	0.69691	1.12114	0.00000
F	-1.37082	2.26758	0.00000
F	1.37082	2.26757	0.00000
F	1.37082	-2.26757	0.00000
F	-1.37082	-2.26758	0.00000

22
TFPyrazine2ndInt.xyz

N	1.61280	0.21490	1.43710
C	1.21210	-0.83210	0.81590
C	0.84570	-0.89720	-0.61370
N	0.95870	0.31250	-1.25950
C	1.38500	1.35780	-0.58860
C	1.71620	1.35230	0.74090
F	2.12760	2.47110	1.41060

F	1.09020	-1.99300	1.52550
F	1.67810	-1.90150	-1.26080
C	-3.88540	0.45060	0.39370
C	-3.38710	0.64220	-0.89560
C	-2.22740	-0.01270	-1.30690
C	-3.22070	-0.40710	1.26990
C	-2.06100	-1.06670	0.86410
C	-1.56270	-0.86880	-0.42590
H	-4.78820	0.96200	0.71090
H	-3.90090	1.30570	-1.58410
H	-1.82610	0.12990	-2.30290
H	-3.60440	-0.56550	2.27270
H	-1.53750	-1.73860	1.53480
O	-0.44430	-1.55980	-0.84430
F	1.47830	2.52090	-1.28920

22
TFPyrazineFirstInt.xyz

N	1.36310	0.10890	1.37800
C	1.53690	-0.97220	0.66880
C	1.68760	-0.91550	-0.72060
N	1.66910	0.22420	-1.35740
C	1.48000	1.31370	-0.64130
C	1.33210	1.25800	0.73250
F	1.15730	2.37340	1.44840
F	1.59670	-2.14090	1.30330
F	1.92330	-2.02370	-1.41350
C	-3.80530	0.94450	0.48070
C	-3.29420	0.92850	-0.82350
C	-2.31210	0.02180	-1.20320
C	-3.30270	0.01290	1.39760
C	-2.31920	-0.89900	1.03350
C	-1.76580	-0.94630	-0.29170
H	-4.57390	1.65450	0.77040
H	-3.67080	1.64100	-1.55570
H	-1.92670	0.02280	-2.22110
H	-3.68550	0.00280	2.41680
H	-1.93880	-1.61650	1.75790
O	-0.85310	-1.78280	-0.63020
F	1.44180	2.48470	-1.28440

22
TFPyrazineTransitionState.xyz

N	1.55638	0.16162	1.43302

C	1.46741	-0.93155	0.75788
C	1.21457	-0.97281	-0.66189
N	1.30010	0.18792	-1.31984
C	1.37230	1.29690	-0.60894
C	1.49580	1.30973	0.75650
F	1.59048	2.46185	1.46545
F	1.55187	-2.10503	1.42110
F	1.74870	-2.04535	-1.32367
C	-3.60484	0.77694	0.44112
C	-3.09523	0.88327	-0.85533
C	-2.03894	0.07688	-1.27107
C	-3.04344	-0.15445	1.31801
C	-1.98568	-0.96271	0.91009
C	-1.45982	-0.87198	-0.39858
H	-4.43117	1.40409	0.76030
H	-3.52582	1.60129	-1.54812
H	-1.64495	0.15404	-2.27913
H	-3.43261	-0.25004	2.32807
H	-1.55054	-1.69154	1.58768
O	-0.46553	-1.66322	-0.79468
F	1.37352	2.45962	-1.29736

22
TFPyridazine3firstINT.xyz

C	1.45280	0.74810	0.49140
C	1.57290	-0.61410	0.53660
C	1.56820	-1.27680	-0.70380
N	1.46560	-0.68850	-1.85760
N	1.33940	0.65630	-1.90000
C	1.33390	1.32210	-0.78570
F	1.21390	2.64990	-0.86480
F	1.45390	1.49710	1.59190
F	1.75520	-1.26390	1.67510
F	1.72780	-2.59720	-0.70620
C	-3.72930	1.27420	0.17130
C	-3.59000	0.32830	-0.85330
C	-2.63410	-0.67690	-0.78150
C	-2.87520	1.18130	1.27610
C	-1.91200	0.18170	1.36200
C	-1.74430	-0.81300	0.33870
H	-4.48010	2.05600	0.11030
H	-4.24130	0.38080	-1.72420
H	-2.54040	-1.40610	-1.58420
H	-2.96280	1.90490	2.08480
H	-1.26100	0.12220	2.23220

O	-0.86530	-1.74570	0.41560

21
TFPyridazine3Prod.xyz

C	2.77334	0.26185	0.00005
C	1.62896	1.00966	0.00001
C	0.39819	0.31139	-0.00012
N	0.32854	-1.00166	-0.00021
N	1.45710	-1.73168	-0.00017
C	2.60907	-1.13450	-0.00005
F	3.70431	-1.89642	-0.00002
F	3.97884	0.81675	0.00017
F	1.66383	2.33671	0.00010
C	-4.53428	-0.59026	0.00018
C	-3.88922	-0.32480	1.20800
C	-2.59878	0.20294	1.21416
C	-3.88974	-0.32410	-1.20775
C	-2.59930	0.20364	-1.21416
C	-1.97506	0.45596	-0.00006
H	-5.53906	-0.99840	0.00028
H	-4.38900	-0.52574	2.14929
H	-2.08373	0.42153	2.14253
H	-4.38993	-0.52449	-2.14894
H	-2.08464	0.42277	-2.14262
O	-0.70937	1.07421	-0.00020

22
TFPyridazine3secondINT.xyz

C	1.74960	0.90190	0.44840
C	1.26340	-0.33800	0.67290
C	0.69010	-1.12210	-0.42390
N	0.62170	-0.49930	-1.66110
N	1.14610	0.72510	-1.84060
C	1.67380	1.39340	-0.87380
F	2.15980	2.64130	-1.13010
F	2.27530	1.65260	1.43610
F	1.28070	-0.87640	1.91660
F	1.46440	-2.36960	-0.52620
C	-3.90610	0.73220	0.41240
C	-3.59920	0.19800	-0.83970
C	-2.47860	-0.61470	-1.00420
C	-3.08790	0.44370	1.50450
C	-1.96630	-0.36940	1.34650
C	-1.66010	-0.89990	0.09110

H	-4.77910	1.36470	0.53650
H	-4.23310	0.41560	-1.69350
H	-2.22280	-1.03120	-1.97030
H	-3.32100	0.85190	2.48290
H	-1.32290	-0.60160	2.18780
O	-0.58190	-1.74930	-0.04760

22
TFPyridazine3TS.xyz

C	1.68531	0.85434	0.49550
C	1.53331	-0.48314	0.64357
C	1.03680	-1.24964	-0.47189
N	0.96892	-0.67808	-1.68882
N	1.11548	0.66052	-1.80885
C	1.44297	1.38222	-0.79278
F	1.59238	2.71603	-0.97227
F	2.07531	1.64757	1.50533
F	1.76442	-1.08861	1.82008
F	1.52393	-2.53721	-0.51275
C	-3.59282	1.03691	0.25746
C	-3.34242	0.34478	-0.92976
C	-2.32215	-0.60082	-0.99796
C	-2.80937	0.76580	1.38153
C	-1.78658	-0.17727	1.31973
C	-1.52308	-0.88852	0.12892
H	-4.39106	1.77057	0.30796
H	-3.94736	0.54462	-1.80997
H	-2.12826	-1.14482	-1.91644
H	-2.99580	1.29453	2.31227
H	-1.17891	-0.39430	2.19342
O	-0.56071	-1.81195	0.07838

22
TFPyridazine4firstINT.xyz

N	1.21300	-0.62010	1.80120
C	1.51670	-1.16480	0.66230
C	1.66860	-0.46700	-0.54910
C	1.49070	0.88990	-0.47960
C	1.16050	1.41590	0.77680
N	1.03530	0.71760	1.86660
F	1.70380	-2.48490	0.64930
F	2.08660	-1.06720	-1.65120
F	1.60160	1.67020	-1.55370
F	0.97130	2.73500	0.87140

C	-3.82390	0.62000	0.53330
C	-3.00470	1.26310	-0.40100
C	-1.93420	0.60450	-0.99770
C	-3.54090	-0.71420	0.85510
C	-2.47360	-1.38370	0.27160
C	-1.61200	-0.76230	-0.69500
H	-4.65950	1.13890	0.99280
H	-3.20660	2.29890	-0.66850
H	-1.31430	1.11710	-1.73050
H	-4.16510	-1.23610	1.57830
H	-2.26530	-2.41980	0.53050
O	-0.62560	-1.38150	-1.23740

21
TFPyridazine4Prod.xyz

N	-3.09986	0.97084	0.32852
C	-1.88667	1.38749	0.12283
C	-0.77063	0.57403	-0.19363
C	-1.06713	-0.77091	-0.29208
C	-2.39212	-1.15805	-0.04374
N	-3.36452	-0.34489	0.25067
F	-1.67939	2.70137	0.21274
F	-0.15433	-1.68445	-0.61091
F	-2.68652	-2.45552	-0.12479
C	4.10638	-0.55184	0.42208
C	3.86607	0.05012	-0.81275
C	2.61663	0.59846	-1.09696
C	3.09536	-0.59745	1.38150
C	1.84295	-0.04685	1.11448
C	1.62434	0.53478	-0.12840
H	5.07915	-0.97937	0.63811
H	4.64923	0.09176	-1.56164
H	2.40647	1.06870	-2.05032
H	3.27895	-1.05593	2.34691
H	1.05914	-0.06269	1.86346
O	0.40610	1.17307	-0.42802

22
TFPyridazine4secondINT.xyz

N	1.43010	-0.53260	1.88220
C	1.19350	-1.11310	0.76250
C	1.02240	-0.53470	-0.58090
C	1.21120	0.88740	-0.42940
C	1.45020	1.44510	0.79790

N	1.57290	0.81740	1.95500
F	1.05300	-2.47290	0.80050
F	1.96190	-1.12410	-1.50750
F	1.08480	1.67040	-1.54670
F	1.57750	2.79390	0.86400
C	-3.87670	0.19050	0.33380
C	-3.18650	1.05360	-0.51770
C	-1.94750	0.68560	-1.03960
C	-3.32300	-1.04760	0.65960
C	-2.08320	-1.42190	0.14230
C	-1.39800	-0.55450	-0.70940
H	-4.84120	0.47940	0.73790
H	-3.61210	2.01750	-0.77760
H	-1.40030	1.34790	-1.69990
H	-3.85510	-1.72540	1.31920
H	-1.63980	-2.38030	0.38810
O	-0.19930	-0.95570	-1.27100

22
TFPyridazine4TS.xyz

N	1.37644	-0.56744	1.85405
C	1.47447	-1.15580	0.70932
C	1.39842	-0.54256	-0.58000
C	1.41561	0.85075	-0.48282
C	1.28983	1.42477	0.77280
N	1.27179	0.78405	1.91507
F	1.61051	-2.49694	0.72401
F	2.03587	-1.13716	-1.61506
F	1.45393	1.60894	-1.59967
F	1.20918	2.76628	0.84580
C	-3.70859	0.46649	0.46054
C	-2.92716	1.22740	-0.41107
C	-1.77744	0.69245	-0.98809
C	-3.32004	-0.84544	0.74935
C	-2.16955	-1.38489	0.18399
C	-1.37015	-0.63730	-0.71636
H	-4.60567	0.88561	0.90502
H	-3.21696	2.24841	-0.64528
H	-1.18003	1.28369	-1.67527
H	-3.91771	-1.44918	1.42726
H	-1.86510	-2.40335	0.40879
O	-0.29626	-1.16585	-1.27326
